# Sensor-Based Differential Flatness-Based Trajectory Tracking Method and Its Application to Wheeled Mobile Robot Control

**DOI:** 10.3390/s26123676

**Published:** 2026-06-09

**Authors:** Alexander Krasavin, Gaukhar Nazenova, Adema Dairbekova, Albina Kadyroldina, Tamás Haidegger, Darya Alontseva

**Affiliations:** 1 School of Digital Technologies and Artificial Intelligence, D. Serikbayev East Kazakhstan Technical University, 19 Serikbayev Street, Ust-Kamenogorsk 070010, Kazakhstan; akrassavin@ektu.kz (A.K.); nazenovagaukhar@gmail.com (G.N.); dairbekova.adema@gmail.com (A.D.); kadyroldina_at@enu.kz (A.K.); 2 Department of System Analysis and Management, L.N. Gumilyov Eurasian National University, Satpayev 2, Astana 010008, Kazakhstan; 3 University Research and Innovation Center (EKIK), Obuda University, Bécsi út 96/b, 1034 Budapest, Hungary

**Keywords:** wheeled mobile robot (WMR), nonlinear control, trajectory tracking, differential flatness, computed torque control (CTC), smart mobility programming

## Abstract

This article investigates the trajectory tracking control of a differential-drive two-wheeled mobile robot (DDWMR) using its kinematic model, framed in the context of sustainability. A nonlinear-to-linear transformation based on differential flatness is employed to convert the original nonlinear system into two fully decoupled linear subsystems, enabling a simple and robust controller design. Unlike conventional flatness-based methods that rely on exact feedforward linearization around a reference trajectory, the proposed approach performs plant linearization, ensuring reliable tracking across a wide range of trajectories. The resulting two-loop architecture consists of an inner nonlinear loop implementing state prolongation and static feedback and an outer linear controller performing trajectory tracking of the linearized system. Simulation results on a circular reference trajectory demonstrate high tracking accuracy, with a maximum transient deviation of 0.28 m, a settling time of approximately 120 s, and a steady-state mean tracking error below 0.01 m. These results confirm that the plant-linearization-based framework provides accuracy, robustness, and practical applicability for DDWMR trajectory tracking, all within a responsible control environment.

## 1. Introduction

Mobile robot control has been an active research topic for the past three decades due to a wide range of practical applications, including mining, logistics and transportation, planetary exploration, automated welding, and agriculture [1,2,3,4]. Beyond the underlying algorithms and hardware, modern mobile robotics is increasingly shaped by sustainability and Environmental, Social, and Governance (ESG) responsibility requirements at the application level. Sustainability in mobile robot control constitutes an integrated control paradigm in which energy consumption, lifecycle resource utilization, and environmental impact are explicitly embedded into the control objectives and constraints, thereby transforming classical feedback control into a multi-objective, resource-aware optimization problem that ensures long-term operational efficiency and ecological compatibility without compromising system stability or performance. International standards also support it now, e.g., IEEE 7000:2022 provides process-oriented guidelines for value-driven, ethically sound design throughout the product lifecycle: from stakeholder value identification to requirements for the design, validation, and field operation of robotic systems [5,6]. The contribution of mobile robots has also been mapped to the United Nations Sustainable Development Goals (SDGs): improving the efficiency and safety of work, logistics, and manufacturing (SDGs 8, 9, 11); energy-efficient motion planning and resource-aware operation (battery usage, wear, maintenance) (SDGs 7, 12, 13); environmental and industrial safety monitoring (SDGs 3, 6, 13); and inclusive human–robot interaction and service accessibility (SDGs 10, 11) [7].

Our study explicitly takes these considerations into account: the chosen speed and heading control scheme allows for the integration of sustainability metrics (cycle energy consumption, braking/acceleration intensity, predicted drive wear) into the controller’s objective functions and constraints while maintaining the traceability of requirements: from value and risk levels to reference signal filters and compensation channels. This strengthens the justification for the chosen compensation and multi-channel control algorithms aimed at improving the reliability and safety of wheeled mobile robot (WMR) operation.

A distinctive feature of many mobile robotic systems is the presence of non-integrable (i.e., nonholonomic) kinematic constraints [8]. This by itself significantly complicates control synthesis; in addition, for nonholonomic mobile robots, the necessary condition for smooth stabilization (Brockett’s condition) is often not satisfied [9], which makes trajectory tracking and path following particularly challenging. In recent years, a wide range of methods have been proposed and applied to nonholonomic mobile robots, including sliding mode control [10,11,12,13], the backstepping method [14], and Nonlinear Model Predictive Control (NMPC) [15,16].

A rapidly developing area of research worth noting is the control of wheeled mobile manipulators (WMMs). While this field was previously dominated by “stop-and-grab” strategies, where the base of the manipulator stops before the arm executes the grasp, operations requiring simultaneous locomotion and manipulation have recently been actively explored [17,18]. An example of a complex problem of this kind is door opening using a WMM, which is discussed in the paper by Xing et al. [18], who propose a promising approach to WMM control that takes into account both positional and orientational compliance, using a controller that adaptively adjusts its parameters based on real-time motion states and estimated interaction torques and forces.

### 1.1. Differential Flatness-Based Trajectory Tracking Control

Among the trajectory tracking methods for nonlinear control systems, approaches based on differential flatness play a special role. If the controlled object has differential flatness, the trajectory tracking problem can be linearized with feedback—that is, a complex nonlinear design problem is reduced to a relatively simple and well-studied linear control problem. Historically, differentially based flatness theory arose from trajectory planning and tracking problems; interest in flatness-based control remains since flat systems have properties that make the generation and implementation of trajectory tracking control particularly efficient. Today, both trajectory planning based on differential flatness [19,20,21] and trajectory tracking control methods [22,23,24] for various types of mobile robots are the subject of intensive research due to their natural advantages over methods that do not exploit the flatness property of the controlled nonlinear object. The assertion of the superiority of flatness-based control over competing methods is supported by experimental results. Sun et al. conducted a comparative study of one of the most popular control methods for mobile robots, Model Predictive Control (MPC), and the Agile Flight method for flat systems control of quadcopters and concluded that control quality is superior with flatness-based methods [25]. Moreover, as recent research results show, differential flatness, as a property of a nonlinear control system, can be effectively used to implement various control methods, such as optimal control [26], Kalman filter-based methods [27], and even model-free control methods [28].

Clearly, not every control system relies on flatness. A flat system is characterized by the existence of a flat (linearizing) output, such that at any instant in time, the state vector and control actions are algebraic functions of this output and a finite number of its time derivatives. Intuitively, by observing only the flat output, one can reconstruct the state and input without direct measurement. The corresponding mappings act as an inverse model of the nonlinear plant, which explains the similarity between flatness-based controllers and inverse model-based design; in practice, flatness is established constructively by exhibiting a suitable flat output as a function of the state, the input, and a finite number of derivatives of the input. Thus, differential flatness is a property of a particular control system, although sometimes the system can be modified to obtain a flat output [29,30].

For WMR, several classes are known that are differentially flat under the no-slip condition [31] or under appropriate inertia distributions for some non-underactuated mobile manipulators [32]. In the case of differential drive, a common choice of flat output signal is the time trajectory of the midpoint of the axle connecting the drive wheels.

### 1.2. Differential Flatness-Based Trajectory Control for WMR

Several differential flatness-based trajectory tracking control methods have been proposed and experimentally validated for WMR with differential drive [33,34,35]. In general, most of the currently known flatness-based trajectory control methods for WMR implement a concept known as exact feedforward linearization. This concept allows designing the control of nonlinear differential flat systems as a specific combination of a nominal feedforward input and a simple stabilizing feedback control. The results of Hagenmeyer and Delalo [36] transform the exact feedforward linearization based on differential flatness into a general control methodology for flat systems. An excellent explanation of the application of this general methodology to the trajectory tracking control problem of a wheeled mobile robot can be found in [37]. One of the advantages of the method under consideration (meaning the exact feedforward linearization method) is that the feedforward linearizing input signal in an open-loop system can practically lead to a linear system in Brunovsky form, thereby reducing the control problem of a nonlinear system (possibly with nonholonomic constraints) to the control problem of a simple linear system in canonical form. Experience has shown that the feedforward linearization method provides a good local solution for the control and trajectory tracking control of a large class of nonlinear systems.

At the same time, the use of exact feedforward linearization is precisely the way to approximately linearize a trajectory tracking control system. It should be noted that theoretically, any differentially flat control system can be linearized by means of coordinate transformation and (in general) dynamic feedback [38]; conversely, any nonlinear system that can be linearized by means of dynamic feedback is flat [39]. We emphasize that the term “linearization of differentially flat control systems” usually means the transformation of a nonlinear system into an equivalent trivial linear control system. Clearly, if such a linearization procedure can be found for a given control system, then a trajectory tracking controller can be constructed on this basis that significantly outperforms (in both robustness and performance) a controller using exact feedforward linearization.

Although flatness theory for control systems mainly developed in the 1990s (e.g., Fliess et al. [40]), practical trajectory tracking methods for nonlinear flat systems predate these theoretical developments. A prominent example is computed torque control (CTC), also known as inverse dynamics control, for sequential manipulators. In fact, CTC implements a static feedback linearization that transforms a nonlinear control system into a trivial linear control system, allowing the design of standard linear trajectory tracking controllers with excellent performance and comparatively simple implementation. Remarkably, nonlinear flat control systems, such as the kinematic and dynamic models of a differentially driven (or differential drive) wheeled mobile robot (DDWMR), share a key feature with serial manipulators: the flat output coincides with the state vector component, which greatly simplifies the linearization of these control systems.

The design of the trajectory tracking controller proposed in this paper is closely related to the CTC methods, and the linearization procedure used in it is a kind of analogue of the static linearization of feedback used both in the CTC and modern methods of robust and adaptive control of torque-controlled serial manipulators and control systems similar to manipulators of this type [41,42,43].

The authors of this paper have previously applied both inversion-based modeling methods [44] and endogenous linearization-based tracking control of a DDWMR model [45] to control a wheeled mobile robot, as well as differential geometry methods to control a manipulator [46,47,48]. In this paper, the CTC and CTC-like methods have been identified as control methods for a special type of flat system, which is referred to herein as a flat system of type A. This type of flat system is characterized by its flat output being a function of the system state only. Specifically, serial manipulators, DDWMRs, and many other mobile robots are flat systems of this type.

The goal of our study was to develop a unified method of trajectory control of flat-type A systems, while we consider the classical method of CTC as a special case of this unified approach. A theoretical generalization of the proposed approach is a class of coordinate transformations of control systems, which we call class C coordinate transformations, each of which corresponds to a static feedback transformation that preserves the trajectory equivalence of the systems. This paper presents a framework for control systems on manifolds that allows us to determine, for two given control systems, whether a transformation of class C connecting these systems exists and to generate it when positive. If one of the control systems is locally a trivial linear system, then this framework allows one to determine the system’s flat output and to synthesize a linearizing transformation. The synthesis of a DDWMR trajectory tracking controller is given here as an example of applying the general method.

### 1.3. Relevance to Sustainable Development Goal 11 (Sustainable Cities and Communities)

Smart and sustainable urban mobility refers to “a modern approach to urban transport that aims to improve the flow of people and goods, reduce congestion and environmental impact, while increasing efficiency, accessibility, and user convenience” (https://www.sciencedirect.com/topics/social-sciences/smart-urban-mobility, accessed on 1 December 2025). It builds on advanced communication systems [38] and clear, high-quality control solutions for mobile robots in the smart city ecosystem. Reducing the latter, an appropriate linearization and trajectory control framework contribute to SDG 11 while strengthening autonomous mobile robotic systems that can support the sphere and create more efficient, more sustainable urban environments. DDWMRs equipped with energy-aware control architectures are suitable for logistics, inspection, mobility, and monitoring tasks in smart city infrastructures. To achieve reliability, resource efficiency, and functional safety, the development of specialized, technologically integrated, and sustainable cities requires a method for their development. Similar solutions should be developed for smart urban aerial mobility systems [49].

## 2. Static Feedback Linearization and Flat Control Systems of Type A and Trajectory Control of Type A Flat Systems

### 2.1. Statement of the Problem, Preliminary Discussion, and Basic Definitions

First of all, it is important to note that a differentially flat control system is sometimes defined as a control system with a so-called flat output $z\left(t\right)$, characterized by the properties given below.

Let us denote $x\left(t\right)$ as the state of a flat control system and $u\left(t\right)$ as the value of the control signal at a given time t. The output $z\left(t\right)$ is a flat output if there exist two functions such that for any t, Equation (1) hold

(1) $$\begin{array}{cc} x\left(t\right)=\alpha \left(z\left(t\right),\dot{z}\left(t\right),\ldots ,z^{\left(p\right)}\right) & u\left(t\right) \end{array}=\beta \left(z\left(t\right),\dot{z}\left(t\right),\ldots ,z^{\left(p+1\right)}\left(t\right)\right)$$

In general, the flat output depends on the current state of the system and the current values of the control signal, as well as the first m time derivatives of the control signal. That is, the flat output z is defined by an algebraic function of the arguments according to Formula (2):

(2) $$z\left(t\right)=F\left(x\left(t\right),u\left(t\right),\dot{u}\left(t\right),\ddot{u}\left(t\right),\ldots ,u^{\left(k\right)}(t)\right)$$

Although from a theoretical point of view, the existence of a flat output is a property of a flat system, which is derived from its definition, most known methods of differential flatness-based control of DDWMRs rely on the above definition. There are good reasons for this. In practice, estimating whether a given nonlinear control system is a differentially flat control system means finding the flat output signal of the system. Moreover, in some cases, the only reason to call a control system differentially flat is its flat output signal, particularly in the case of DDWMRs. It should be noted that for some applications, this simple definition is quite sufficient and useful. The trajectory of a flat output signal completely determines the trajectory of a flat control system in state space, simplifying trajectory planning for a flat system. Knowing the flat output signal of a control system is sufficient to apply the exact feedforward linearization technique to a nonlinear flat system. However, the very origin of the term “differential flatness” is associated with the fundamental concept of trajectory equivalence of control systems. Any differentially flat control system is trajectory-equivalent to a trivial linear control system, defined below.

**Definition** **1.** *A trivial linear control system $T^{n,k}$ is a linear control system, with control space $U=R^{n}$ and state space $X=X_{1}\times X_{2}\times \ldots {\times X}_{k}$, where $X_{j}\subset R^{n}$ for all 1≤j≤k, with the dynamic equation in the form of a system of k differential Equation (3), for k components of the state vector $x_{1},x_{2},\ldots ,x_{k}$, $x_{j}\in X_{j}$ for all 1≤j≤k.*

(3) $$\begin{array}{c} {\dot{x}}_{1}=x_{2}\\ {\dot{x}}_{2}=x_{3}\\ \begin{array}{c} \ldots \\ {\dot{x}}_{k}=u \end{array} \end{array}\;\;\forall x=\left(x_{1}\times x_{2}\times \ldots \times x_{k}\right)\in X,u\in U$$

In differential flatness theory, a differentially flat control system is defined as a system that is trajectory-equivalent to some trivial linear control system. A fundamental result of differential flatness theory is that any differentially flat control system can be linearized using coordinate transformations and/or dynamic feedback. To avoid misunderstandings regarding this statement, it is necessary to explicitly indicate that the term “linearization” in the context of differential flatness theory means the application of trajectory equivalence-preserving control system transformations to a nonlinear system, the result of which is a trivial linear control system. Trajectory equivalence of control systems is a key concept in differential flatness theory, leading to a highly effective approach to trajectory tracking control of differentially flat systems. A simple analogy can be drawn: just as in physics, equations describing a physical process can have different forms in different coordinate systems; in control theory, the form of a dynamic equation depends (for example) on the choice of coordinates in the state space of the control system. Thus, it may turn out that the choice of coordinate system obscures the fact that we are dealing with a simple linear system. Clearly, rather than attempting to solve a complex nonlinear problem, it would be far better to find a coordinate system in which the problem becomes elementarily simple.

It should be emphasized that until the advent of the concept of exact feedback linearization, first presented in [36], the term “linearization” as applied to flat systems meant exclusively applying a trajectory-equivalence-preserving transformation to a nonlinear system, converting it into a trivial linear system. In the geometric approach to the definition of trajectory equivalence of the control systems used in this paper, coordinate and dynamic feedback transformations of the control systems that preserve trajectory equivalence relate to the so-called endogenous transformations of the corresponding system. Here, the term “system” means a geometric object, namely, a pair consisting of a smooth manifold of infinite dimension and a smooth tangent vector field on this manifold. This is significant, as the transformation linearizing the DDWMR kinematic model belongs to the class of simple endogenous transformations—a coordinate transformation that can be implemented as a partial linearization of static feedback. As will be shown in this paper, any differentially flat system whose flat output depends only on the state can be linearized by applying such a coordinate transformation.

We call the class of differentially flat systems whose flat output depends only on the state of the system class A. An important and best-known example of a type A control system is a serial torque-controlled manipulator. In this paper, a class of trajectory-equivalence-preserving coordinate transformations is presented, which we call class C. Any class C coordinate transformation can be implemented as a static feedback transformation. Therefore, any type A control system can be linearized via static feedback. This allows us to reduce the problem of trajectory control of a nonlinear flat system of type A to a linear problem of trajectory control of a trivial linear system.

In the preliminary discussion, it can be noted that the well-known computed torque control method for serial manipulator trajectory tracking control may be considered an implementation of this approach since the feedback linearization scheme used in the CTC corresponds to a class C coordinate transformation and the resulting linear system is trivial. One of the main contributions of the study presented here is a unified procedure for determining whether a given control system can be linearized using a class C coordinate transformation or not, as well as determining the exact form of this transformation for a system that admits this type of linearization. Moreover, the synthesis of the linearization transformation does not require knowledge of the flat output of the system, and the method for synthesis of the linearization transformation proposed in this paper also provides a method for estimating the flat output of a given system.

### 2.2. Static Feedback Transformation and Coordinate Transformations of the Class C Preserving Trajectory Equivalence

Let B be a control system with state space Y, control space W and dynamic Equation (4)

(4) $$\dot{y}=g\left(y,w\right)\;y\in Y,w\in W$$

Let us consider the static feedback transformation corresponding to the coordinate transformation given by Formula (5):

(5) $$\begin{array}{cc} x=\mu^{-1}\left(y\right) & w= \end{array}\rho \left(x,u\right)$$

where $\mu^{-1}:X\to Y$ is an inverse of the diffeomorphic mapping μ:X→Y and ρ:X×U→W is a smooth mapping satisfying Condition 1.

**Condition** **1.** *For any point p∈X, the mapping $\rho_{p}:U\to W$ defined by Formula (6) is a diffeomorphism.*

(6) $$\forall q\in U\rho_{p}\left(q\right)=\rho \left(\left(p,q\right)\right)\;\left(p,q\right)\in X\times U$$

**Figure 1:**
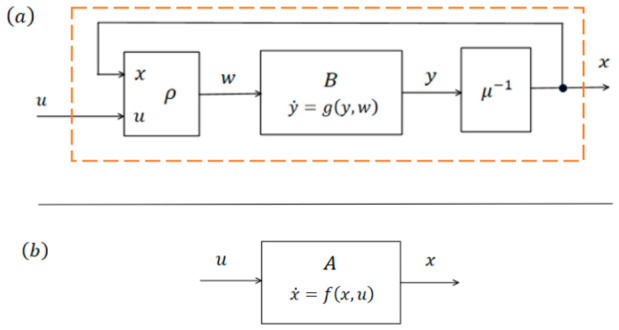
Scheme of transformation by static feedback of the control system B with state space Y and control space W, defined by two mappings μ:X→Y and ρ:X×U→W (**a**), into a control system A with state space X and control space U (**b**).

Figure 1a shows a scheme of such a transformation applied to the control system B.

Let us define the conditions, the fulfillment of which guarantees that the result of the transformation will be a given control system A (Figure 1b), with state space X, control space Y and dynamic Equation (7), trajectory-equivalent to the control system B

(7) $$\dot{x}=f\left(x,u\right)\;x\in X,u\in U$$

The static feedback transformation, the scheme of which is shown in Figure 1, is completely determined by the mappings μ and ρ. At the beginning, we will consider the control system A simply as the result of this transformation applied to the given control system B. Let $\gamma \left(t\right)=\left(x_{\gamma }\left(t\right),u_{\gamma }\left(t\right)\right)$ be an arbitrary trajectory of the control system A. Then, curve $\sigma \left(t\right)=\left(\mu \left(x_{\gamma }\left(t\right)\right),\rho \left(x_{\gamma }\left(t\right),u_{\gamma }\left(t\right)\right)\right)$ on Y×W is the trajectory of the control system B.

It would be convenient to consider mappings μ and ρ as components of mapping h:X×U→Y×W, defined by Formula (8)

(8) $$x\in X,u\in U\;h\left(x,u\right)=\left(\mu \left(x\right),\rho \left(x,u\right)\right)\;$$

Since for an arbitrary trajectory $\gamma \left(t\right)$ of the control system A, $\sigma \left(t\right)=h\left(\gamma \left(t\right)\right)$ is a trajectory of the system B, the mapping h:X×U→Y×W defines a one-to-one mapping of the set of trajectories of the system A onto the set of trajectories of the system B. Consequently, the mapping h must be invertible or, more precisely, h must be a diffeomorphic mapping, so that the system A can be trajectory-equivalent to the original system B. Since the mapping μ is diffeomorphic and, for a smooth mapping ρ, Condition 1 is satisfied, the inverse mapping exists and is defined by Formula (9).

(9) $$\forall q=\left(y,w\right)\in Y\times W\;h^{-1}\left(q\right)=\left(\mu^{-1}\left(y\right),\rho^{†}\left(y,w\right)\right)$$

where mapping $\rho^{†}:Y\times W\to U$ dual to mapping ρ:X×U→W, is defined by Formula (10)

(10) $$\forall q=\left(y,w\right)\in Y\times W\;\rho^{†}\left(q\right)=\rho_{\mu^{-1}\left(y\right)}^{-1}\left(w\right)$$

**Figure 2:**
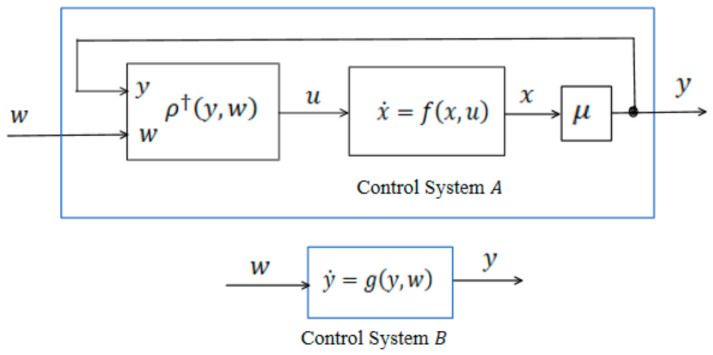
A static feedback transformation corresponding to the inverse mapping $h^{-1}$, converting the control system A into the control system B.

The mapping $h^{-1}$ defines the inverse of a given h, a static feedback transformation, the schematic of which is shown in Figure 2.

Now we can determine the dynamic equation of the system A analytically from the equation of the dynamics of the system B**.** We will interpret the signals $x\left(t\right)$ and $y\left(t\right)$ as components of some trajectory $\left(x\left(t\right),y\left(t\right)\right)\;$of the control system A, and $y\left(t\right)=\mu \left(x\left(t\right)\right)$ as a trajectory in the state space Y of the control system B. Differentiating the last equation with respect to time, we obtain Equation (11):

(11) $$\dot{y}=d_{x}\mu \left(\dot{x}\right)$$

From this follows Equation (12) for the vectors of the tangent spaces ${f\left(x,u\right)\in T}_{x}X$ and ${g\left(y,w\right)\in T}_{\mu \left(x\right)}Y$:

(12) $$g\left(y,w\right)=d_{x}\mu \left(f\left(x,u\right)\right)$$

Equation (9) corresponds to the dual Equation (13) in the tangent space $T_{y}Y$:

(13) $$f\left(x,u\right)=d_{y}\mu^{-1}\left(g\left(y,w\right)\right)$$

Equations (9) and (10) are valid at any point $\left(x(t),y(t)\right)\in M$ and $h\left(y,w\right)\in N$ of the trajectories of the systems A and B. Since some trajectory of the system A passes through any point X×U and, by definition, the mappings h:X×U→Y×W and $h^{-1}:Y\times W\to X\times U\;$are surjective, the general Equations (14) and (15) are valid, each of which represents a condition of trajectory equivalence of the systems A and B:

(14) $$\forall p=\left(x,u\right)\in X\times U\;d\mu_{x}\left(f\left(x,u\right)\right)=g\left(\mu \left(x\right),\rho \left(x,u\right)\right)\;$$

(15) $$\forall q=\left(y,w\right)\in Y\times W\;d\mu_{y}^{-1}\left(g\left(y,w\right)\right)=f\left(\mu^{-1}\left(y\right),\rho^{†}\left(y,w\right)\right)$$

From Formula (15) follows Formula (16), which analytically determines the function f from a given function g:

(16) $$\forall x\in X,u\in U\;f\left(x,u\right)=d_{\mu \left(x\right)}\mu^{-1}\left(g\left(\mu \left(x\right),\rho \left(x,u\right)\right)\right)$$

**Figure 3:**
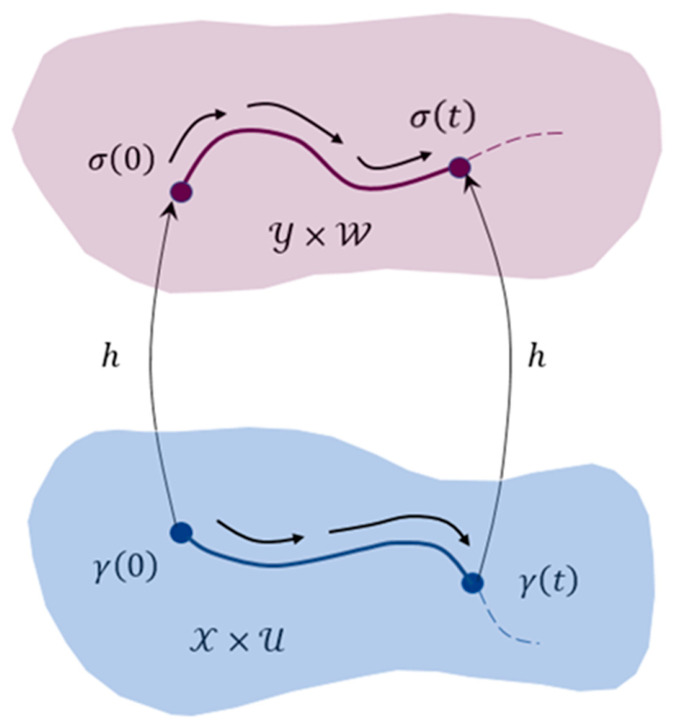
Correspondence of trajectories of equivalent control systems related by a coordinate transformation of class C, given by a diffeomorphic mapping h: X×U→Y×W. For any trajectory γ of control system A, the smooth curve σ on Y×W given by the equation $\sigma \left(t\right)=h\left(\gamma \left(t\right)\right)$ is a trajectory of control system B.

Note that within the framework of the geometric approach to defining the concept of trajectory equivalence of control systems presented in paper [38], Formula (12) represents the condition that the mapping h:X×U→Y×W must satisfy in order for the given control systems A and B, related by the coordinate transformation specified by the mapping h, to be trajectory-equivalent to each other. We will call such coordinate transformations of control systems class C coordinate transformations. Note that in the case under consideration, the concept of trajectory equivalence of control systems allows for a clear interpretation (see Figure 3) and is self-explanatory in nature.

Note that the trajectory equivalence condition (14) can be viewed as an equation for the unknown function $\rho \left(x,u\right)$. Section 3 describes a new algorithm that, given two control systems, determines whether a class C transformation exists to transform the control system A into the control system B and, if such a transformation is theoretically feasible, identifies it. Since any class C coordinate transformation defines a physically implemented static feedback transformation, we refer to the application of this algorithm as trajectory controller synthesis and provide examples.

Now suppose that the result of the transformation of class C applied to a nonlinear system B, which we call the system A, is the trivial linear system $T^{n,k}$. In this case, the system B is, by definition, a differentially flat control system. Below, we define the flat output of the system B and the functions α, β (1) that characterize B as a flat system, from the components μ:X→Y and ρ:X×U→W of the mapping h:X×U→Y×W. By the definition of the trivial linear system $T^{n,k}$, the state space of the system A is the Cartesian product of k components $X=X_{1}\times X_{2}\times \ldots \times X_{k}$. Therefore, mapping $\mu^{-1}:Y\to X$ can be represented by k—components $\varphi_{j}:Y\to X_{j}$, such that, for an arbitrary y∈Y, the identity $\mu^{-1}\left(y\right)=\left(\varphi_{1}\left(y\right),\varphi_{2}\left(y\right),\ldots ,\varphi_{k}\left(y\right)\right)$ holds. The component $h_{1}:X\to R^{n}$ of the mapping $\mu^{-1}:Y\to X$ defines the flat output $z\left(t\right)$ of the nonlinear control system B according to Equation (17)

(17) $$\forall t\;z\left(t\right)=h_{1}\left(y\left(t\right)\right)$$

Note that the flat output $z\left(t\right)$ of a control system B depends only on the current state $y\left(t\right)$. We call the class of systems whose flat output is a function of the state flat control systems of type A since any state x∈X of a control system A can be represented in the form $x=\left(x_{1},x_{2},\ldots {,x}_{k}\right)$, where the relationship between the algebraic function of k arguments α and the mapping μ:X→Y can be represented by Equation (18), and the relationship between the function β and the mapping and ρ:X×U→W in the form (19)

(18) $$\forall x=\left(x_{1},x_{2},\ldots ,x_{k}\right)\in X\;\;\mu \left(x\right)=\alpha \left(x_{1},x_{2},\ldots ,x_{k}\right)$$

(19) $$\forall \left(x_{1},x_{2},\ldots ,x_{k}\right)\in R^{nk},u\in R^{n}\;\;\rho \left(\left(x_{1},x_{2},\ldots ,x_{k}\right),u\right)=\beta \left(x_{1},x_{2},\ldots ,x_{k},u\right)$$

As follows from Equations (18) and (19), if the flat output for a given type A flat system is known (which implies that the functions are also known), then mappings defining a linearizing feedback transformation can be found from them. Regardless of how the class C coordinate transformation linearizing a given type A flat system is constructed, this transformation defines a simple but effective trajectory tracking controller for this system, as described in the next section.

### 2.3. Unified Trajectory Tracking Controller for Type a Flat Control Systems

**Figure 4:**
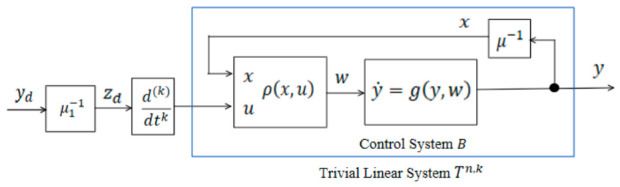
Basic feedforward trajectory control scheme for a flat type A system, trajectory-equivalent to the trivial linear control system $T^{n.k}$.

In the case where a flat control system B of type A is trajectory-equivalent to a trivial linear system $T^{n.k}$, then by applying to this system a static feedback transformation defined by the function $\rho \left(x,u\right)$, associated with the function β of the flat control system B by the identity relation (16) and the function μ, identical to the function α of this system, we reduce the problem of trajectory control of the nonlinear system B to the problem of trajectory control of the linear system $T^{n.k}$. Formally, this means that if $y_{0}\in Y$ is the initial state of the system B, subjected to transformation by static feedback, then for any given trajectory of the control signal $u\left(t\right)$ on the time interval $t\in \left[0,\infty )\right.$, the corresponding trajectory in the phase space y(t) of the system B is determined by the equality $\forall t\in \left[0,\infty )\right.\;y\left(t\right)=\mu^{-1}\left(x\left(t\right)\right)$, where $\left(u\left(t\right),x\left(t\right)\right)$ is the trajectory of the “virtual” trivial linear system $T^{n.k}$ determined by the initial state $x\left(0\right)=\mu^{-1}\left(y_{0}\right)$ and the given trajectory of the control signal $u\left(t\right)$. Obviously, in order to completely specify the desired trajectory $\left(x_{d}\left(t\right),u_{d}\left(t\right)\right)$ of the trivial linear system $T^{n.k},\;$it is sufficient to specify its initial state and the trajectory $x_{d}^{1}\left(t\right)$ of the first component of the state vector $x_{d}\left(t\right)=\left(x_{d}^{1}\left(t\right),x_{d}^{2}\left(t\right),\ldots ,x_{d}^{2}\left(t\right)\right)$, since for any trajectory of the trivial linear system $T^{n.k},$ the identity $\forall t\;u\left(t\right)=\frac{d^{\left(k\right)}}{dt^{k}}x_{1}\left(t\right)$. Thus, we arrive at the feedforward trajectory tracking controller scheme shown in Figure 4.

Note that an alternative feedforward controller is possible, in which the flat output of the system and its time derivatives act as the state feedback signal. Still, it does not provide any advantage over the basic scheme shown in Figure 4, so in the future, it will be presented only as an intermediate option when describing the synthesis of the DDWMR trajectory tracking controller.

Since the trajectory of the flat output completely determines the trajectory in the state space of the flat system, it is reasonable to take the desired trajectory of the flat output $\mu^{-1}\left(t\right)$ as the input of the trajectory control system, i.e., to use the general technique of trajectory control of flat systems—specifying the desired trajectory of the flat output—rather than the desired trajectory of the system as a whole. Moreover, to stabilize the system (organizing state feedback), it is also easier to use a flat output. Thus, in fact, we are constructing a trajectory control system for the flat output of the system, rather than the system state itself. This is the main benefit of using flat systems: they provide such an opportunity.

Moreover, to implement static feedback, in some cases, it is much more advantageous to use the function $d\left(y,u\right)=\rho \left(\mu^{-1}\left(y\right),u\right)$ instead of the function $\rho \left(x,u\right)$.

**Figure 5:**
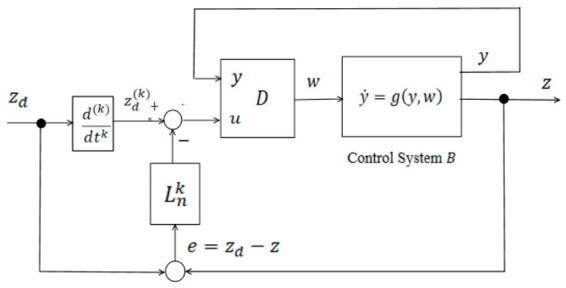
General diagram of the feedback controller of the trajectory control for a flat system of type A, trajectory-equivalent to the trivial linear system with $T^{n.k}$ control. The $L_{n}^{k}$ block is the block of the n-channel linear controller.

Taking this into account, we arrive at the feedback controller circuit for the trajectory control shown in Figure 5.

The L block consists of n identical signal converters connected in parallel. Since our examples will use flat type A systems equivalent to $T^{n.2}$ systems, below, we present the calculation of a linear controller specifically for such systems. Furthermore, it will be demonstrated that the controller’s stability criterion matches the well-known stability criterion for the linear controller included in the CTC manipulator’s trajectory control system.

### 2.4. Description of a Linear Trajectory Control System Equivalent to a Unified Controller for a Second-Order System

**Figure 6:**
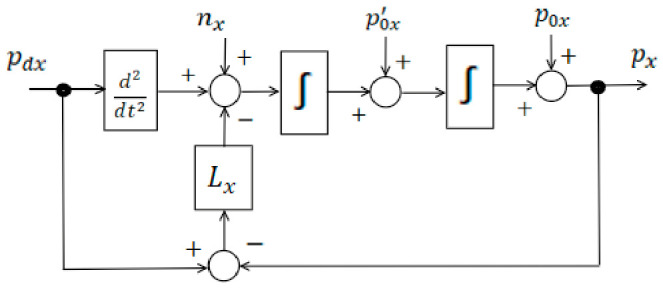
Equivalent block diagram of the x-channel of the trajectory tracking control system for the linearized kinematic model of the DDWMR, where Lx is a single-channel linear signal converter, x is the channel index.

Figure 6 presents the block diagram of the x-channel. The resulting trajectory tracking system can be viewed as two decoupled one-dimensional linear subsystems. We will use integer index x to designate the number of corresponding channels (so $x\in \left\{1,\;2\right\})$.

**Figure 7:**
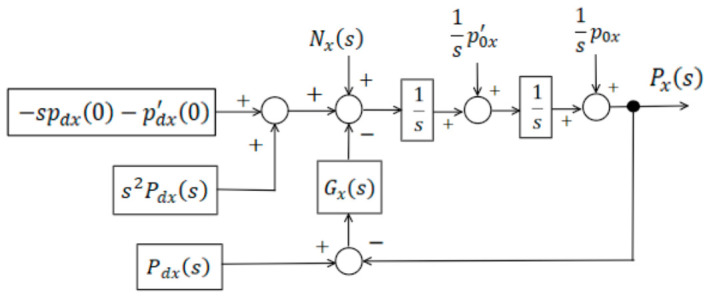
Laplace-domain block diagram of the x-channel of the trajectory tracking control system for the differential-drive mobile robot.

To analyze the behavior of the one-dimensional linear control subsystem, it is convenient to employ the Laplace transform. In this framework, constant functions of the form $f\left(t\right)=c\in R$ are represented as $c\eta \left(t\right)$, where η denotes the Heaviside step function. We use $G_{x}$ to denote the transfer function describing the linear signal transformers $L_{x}$ and $L_{y}$. In addition, we introduce the notations$\;P_{x}\left(s\right)=L\left(p_{x}\left(t\right)\right)$ and $P_{dx}\left(s\right)=L\left(p_{dx}\left(t\right)\right)$. Taking into account that $L\left({\ddot{p}}_{dx}\left(t\right)\right)=s^{2}P_{dx}\left(s\right)-sp_{dx}\left(0\right)-p_{dx}^{\prime }\left(0\right)$ and $L\left(\eta \left(t\right)\right)=\frac{1}{s}$, we obtain, for the Laplace-domain representations of the signals, the block structure describing the behavior of each one-dimensional channel of the robot’s trajectory tracking system, as shown in Figure 7.

The analysis of the one-dimensional linear trajectory tracking subsystem in the Laplace domain leads to expression (20), which describes the relationship between the Laplace transform $P_{x}\left(s\right)$ of the system output component $p_{x}$ and the transform $P_{dx}\left(s\right)$ of the corresponding reference component:

(20) $$P_{x}\left(s\right)=P_{dx}\left(s\right)+E_{x}^{d}\left(s\right)+\frac{N_{x}\left(s\right)}{s^{2}-G_{k}\left(s\right)}$$

where the complex variable function $E_{x}^{d}\left(s\right)$ is defined by expression (21).

(21) $$E_{x}^{d}\left(s\right)=\frac{s\left(p_{0x}-p_{dx}\left(0\right)\right)+\left({p_{0x}^{\prime }-p}_{dx}^{\prime }\left(0\right)\right)}{s^{2}-G_{x}\left(s\right)}$$

Naturally, the function $E_{x}^{d}\left(s\right)$ can be interpreted as the Laplace transform of the time-domain function $e_{x}^{d}\left(t\right)$. As follows from the definition of $E_{x}^{d}\left(s\right)$ in (9), the function $e_{x}^{d}\left(t\right)$ is the solution of the initial value problem for the differential Equation (22) with the initial conditions given in (23).

(22) $${\ddot{e}}_{x}^{d}-Ue_{x}^{d}=0$$

(23) $$\begin{array}{cc} e_{x}^{d}\left(0\right)=p_{0x}-p_{dx}\left(0\right) & {\dot{e}}_{x}^{d}\left(0\right){=p_{0x}^{\prime }-p}_{dx}^{\prime }\left(0\right) \end{array}$$

In the differential Equation (10), the symbol U denotes the linear operator representing the action of the linear signal transformer defined by the transfer function $G_{x}$. Term $\frac{N_{x}\left(s\right)}{s^{2}-G_{x}\left(s\right)}$ in Equation (8) represents the Laplace transform of the disturbance signal $n_{x}\left(t\right)$, passed through a linear filter described by the transfer function $W_{f}\left(s\right)$, defined in expression (24).

(24) $$W_{f}\left(s\right)=\frac{1}{s^{2}-G_{k}\left(s\right)}$$

Thus, the output of the trajectory tracking subsystem $y_{x}\left(t\right)$ is a linear superposition of the three functions of the form (25).

(25) $${\forall t\geq 0\;p}_{x}\left(t\right)=p_{dx}\left(t\right)+e_{x}^{d}\left(t\right)+L^{-1}\left(W_{f}N_{x}\right)$$

As follows from a straightforward analysis of expression (25), the stability of the trajectory tracking system is guaranteed provided that the linear signal transformers $L_{x}$ and $L_{y}$ satisfy Criterion 1, formulated below. It is also evident that in order to minimize the influence of disturbances represented by the term $L^{-1}\left(W_{f}N_{x}\right)$, the superposition (25)—the transfer function ${G=G}_{x}=G_{y}$ of the identical signal transformers $L_{x}$ and $L_{y}$—must satisfy Criterion 2.

**Criterion** **1.** *The linear operator U, corresponding to the identical linear signal transformers $L_{x}$ and $L_{y},$ must stabilize the differential Equation (26).*

(26) $$\ddot{e}-Ue=0\;$$

**Criterion** **2.** *The transfer function G of the linear signal transformers $L_{x}$ and $L_{y}$ must satisfy the disturbance-rejection condition (27).*

(27) $$\forall \omega \geq 0\;\left|G\left(i\omega \right)+\omega^{2}\right|>1$$

As a result, the two stability criteria, achievability of the required dynamic form and satisfaction of the disturbance-rejection condition, ensure the correctness of the constructed transformation and form the foundation for designing the outer trajectory tracking control loop, which will be presented in the next section.

Note that in the first section, the exact state spaces of the described control systems were not specified. The state space of many control systems, including those of great practical interest (manipulators, mobile robots in general), has a complex geometric structure. To describe the state space of such systems, the tool of smooth manifolds is necessary.

## 3. Synthesis of a Trajectory Tracking Controller for Type A Flat Systems

### Synthesis of a Linearizing Transformation That Determines the Controller

Any computational operations involving transformations of control systems on manifolds are performed with local descriptions of these systems. In one way or another, any local description of two control systems on manifolds, explicitly or implicitly, presupposes the existence of two charts that are specifically consistent with each other. Let us introduce a notational convention, the fulfillment of which will be implied in what follows: let $\left(U,\varphi \right)\;U\subset X,\varphi :\;U\to R^{m}$ be a chart on a smooth manifold X describing control system A. Like any other chart of the manifold X, the chart $\left(U,\varphi \right)$ defines a Cartesian basis in $R^{m}$ and the corresponding Cartesian coordinate system.

We denote the orthonormal basis given by $\left(U,\varphi \right)$ in $R^{m}$ as $e=\left\{e_{1},e_{2},\ldots ,e_{m}\right\}$ and the dual basis of e in $R^{m}$ as $e^{\#}=\left\{e^{1},e^{2},\ldots ,e^{m}\right\}$. Any point $x\in R^{m}$ can be represented as a covector $x=\left(x_{1},x_{2},\ldots ,x_{m}\right)=\sum_{i=1}^{m}x_{i}e^{i}$ and as a vector $x={\left[x_{1},x_{2},\ldots ,x_{k}\right]}^{T}=\sum_{i=1}^{m}e_{1}x_{m}.$ A Euclidean vector space with a given basis e is isomorphic to a linear space of covectors with basis $e^{\#}$, and they can be identified. However, vectors and covectors behave differently under nonlinear coordinate transformations, and we will distinguish between these spaces. We will consider the domain $D=\varphi \left(U\right)$ as a domain in the space of covectors in which local coordinates $x_{1},x_{2},\ldots ,x_{m}$ are introduced, and the identical domain of the vector space $R^{m}$ as the tangent space to the manifold D. This corresponds to the fact that the tangent bundle $TR^{m}$ is trivial and there is a natural diffeomorphism $TR^{m}\to R^{m}\times R^{m}$. We will assume that in the control space U of the control system A, coordinates $u_{1},u_{2},\ldots ,u_{n}$ are introduced. For a given u∈U, we interpret $f\left(x_{1},x_{2},\ldots ,x_{m},u\right)\;$as a vector field on D defined as $F_{\left(x_{1},x_{2},\ldots ,x_{m}\right)}$. Thus, given $x=\left(x_{1},x_{2},\ldots ,x_{m}\right)$ and u, $f\left(x,u\right)$ is simply a vector $R^{m}$ that can be represented as $f\left(x,u\right)=$ ${\left[f_{1}\left(x,u\right),f_{2}\left(x,u\right),\ldots ,f_{m}\left(x,u\right)\right]}^{T}$.

Now consider the chart $\left(V,\phi \right)$ on Y, where $V=\mu \left(U\right)$ and $\phi =\mu^{-1}◦\varphi$. We denote M:D→D as the mapping, given by the formula$\;\forall x\in D\;M\left(x\right)=\phi \left(\mu \left(\varphi^{-1}\left(x\right)\right)\right)$. If we work in local coordinates defined by the chart $\left(U,\varphi \right)$, then the equation of the dynamics of the system B is defined in curvilinear coordinates $y_{1},y_{2},\ldots ,y_{m}$ defined by the chart $\left(V,\phi \right)$ in the same region of space D. If the control systems A and B are related by a coordinate transformation of class C, then upon transition to the Cartesian coordinate system defined by the chart $\left(V,\phi \right)$, the equations of the dynamics of the system A become the equations of the dynamics of the system B, and vice versa. This is perhaps the simplest and most intuitive expression of the essence of transformations of this class.

The matrix of the linear operator $d_{x}\mu :TX\to TY$ in Cartesian coordinates, corresponding to the local coordinates $x_{1},x_{2},\ldots ,x_{m},$ will be the matrix of the Jacobian $J\left(x\right)$ of the coordinate transformation $y=M\left(x\right)$, which has the form (28):

(28) $$J\left(x\right)=\left[\begin{array}{cc} \begin{array}{c} \begin{array}{cc} \frac{\partial y_{1}\left(x\right)}{\partial x_{1}} & \frac{\partial y_{1}\left(x\right)}{\partial x_{2}} \end{array}\\ \begin{array}{cc} \frac{\partial y_{2}\left(x\right)}{\partial x_{1}} & \frac{\partial y_{2}\left(x\right)}{\partial x_{2}} \end{array} \end{array} & \begin{array}{c} \begin{array}{cc} \ldots & \frac{\partial y_{1}\left(x\right)}{\partial x_{m}} \end{array}\\ \begin{array}{cc} \ldots & \frac{\partial y_{2}\left(x\right)}{\partial x_{m}} \end{array} \end{array}\\ \begin{array}{c} \begin{array}{cc} \ldots & \ldots \end{array}\\ \begin{array}{cc} \frac{\partial y_{m}\left(x\right)}{\partial x_{1}} & \frac{\partial y_{m}\left(x\right)}{\partial x_{2}} \end{array} \end{array} & \begin{array}{c} \begin{array}{cc} \ldots & \ldots \end{array}\\ \begin{array}{cc} \ldots & \frac{\partial y_{m}\left(x\right)}{\partial x_{m}} \end{array} \end{array} \end{array}\right]$$

Then, the condition of trajectory equivalence of control systems (12) corresponds to the condition in the form of matrix Equation (29):

(29) $$J\left(x\right)f\left(x,u\right)=g\left(M^{-1}\left(x\right),\rho \left(x,u\right)\right)$$

Condition (24) corresponds to a system of m functional Equation (30) for n functions $\rho_{1}\left(x,u\right),\rho_{2}\left(x,u\right),\ldots ,\rho_{n}\left(x,u\right)$, corresponding to the components of the mapping ρ:X×U→W in local coordinates:

(30) $$\left\{\begin{array}{c} \sum_{i=1}^{m}J_{1,i}\left(x\right)\cdot f_{i}\left(x,u\right)=g_{1}\left(x,\rho \left(x,u\right)\right)\\ \sum_{i=1}^{m}J_{2,i}\left(x\right)\cdot f_{i}\left(x,u\right)=g_{2}\left(x,\rho \left(x,u\right)\right)\\ \begin{array}{c} \vdots \\ \sum_{i=1}^{m}J_{m,i}\left(x\right)\cdot f_{i}\left(x,u\right)=g_{m}\left(x,\rho \left(x,u\right)\right) \end{array} \end{array}\right.$$

If the solution of system (30) does not exist, then there is accordingly no transformation of class C that connects the control systems A and B. Otherwise, by solving this equation, i.e., by defining the mapping ρ, we define a transformation of class C and therefore synthesize a transformation by static feedback that transforms the control system B into the control system A.

In some cases, the equations of the dynamics of the control systems A and B can also be given as equations for the so-called state variables of the system. We will consider a special case corresponding to a possible linearization of the control system A. We will assume that the dimension of the state space of the control system A is a multiple of the dimension of the control space and define k state variables $z_{1}=\left(x_{1},x_{2},\ldots ,x_{k}\right),\;z_{2}=\left(x_{k+1},\ldots ,x_{2k}\right),\ldots ,z_{m}=\left(x_{m-k+1},,\ldots ,x_{m}\right)$, so that any state x of the system A can be represented as $x=\left(z_{1},\;z_{2},\ldots ,\;z_{k}\right)$ and the corresponding tangent space vector $x^{T}$ as $x^{T}={\left[z_{1}^{T},z_{2}^{T},\ldots ,z_{k}^{T}\right]}^{T}$. This representation of state vectors corresponds to a local representation of the phase X space of the system in the form of a Cartesian product of covector spaces $Z_{1},Z_{2}\ldots Z_{n}$ of the same dimension $\forall \;dim\;Z_{1}=n$ of the form $X=Z_{1}\times Z_{2}\times \ldots Z_{k}$ and a similar representation of the tangent space $T_{X}={TZ}_{1}\times {TZ}_{2}\times \ldots {TZ}_{k}$.

The mapping M:D→D defines a similar representation of the phase space of the control system $Y{=Y}_{1}\times Y_{2}\times \ldots Y_{k}$*,* where $Y_{j}=M_{j}\left(X\right)$, defining the state variables $y_{1},y_{2},\ldots ,y_{k}$ of the control system Y. Let us consider the case when the equation of the dynamics of the control system A is given by an ODE system of the form (31):

(31) $$\begin{array}{c} {\dot{z}}_{1}=z_{2}\\ \begin{array}{c} {\dot{z}}_{2}=z_{3}\\ \ldots \end{array}\\ {\dot{z}}_{k}=u \end{array}$$

In this case, if the control system B is locally connected to the control system A by a coordinate transformation of class C, then its dynamic equation must have a specific form of a system of differential equations for its components of the type (32):

(32) $$\begin{array}{c} {\dot{y}}_{1}=g_{1}\left(y_{1},y_{2},\ldots ,y_{k}\right)\;\;\;\;\;\\ \begin{array}{c} {\dot{y}}_{2}=g_{2}\left(y_{1},y_{2},\ldots ,y_{k}\right)\;\;\;\;\;\\ \ldots \end{array}\\ {\dot{y}}_{k}=g_{2}\left(y_{1},y_{2},\ldots ,y_{k},w\right) \end{array}$$

Then, for any state vector x of the control system A, the matrix Equation (33), equivalent to Equation (30), but having a simpler form, must be satisfied:

(33) $$\left[\begin{array}{ccc} \frac{\partial y_{1}\left(x\right)}{\partial x_{1}} & \ldots & \frac{\partial y_{1}\left(x\right)}{\partial x_{m}}\\ \vdots & J\left(x\right) & \vdots \\ \begin{array}{c} \frac{\partial y_{m-1}\left(x\right)}{\partial x_{1}}\\ \frac{\partial y_{m}\left(x\right)}{\partial x_{1}} \end{array} & \begin{array}{c} \ldots \\ \ldots \end{array} & \begin{array}{c} \frac{\partial y_{m-1}\left(x\right)}{\partial x_{m}}\\ \frac{\partial y_{m}\left(x\right)}{\partial x_{m}} \end{array} \end{array}\right]\left[\begin{array}{c} z_{2}^{T}\\ \ldots \\ \begin{array}{c} z_{k}^{T}\\ u^{T} \end{array} \end{array}\right]=\left[\begin{array}{c} g_{1}^{T}\left(M_{1}\left(x\right),M_{2}\left(x\right),\ldots ,M_{k}\left(x\right)\right)\;\;\;\;\;\;\;\;\;\;\;\;\;\;\\ \ldots \\ \begin{array}{c} g_{k-1}^{T}\left(M_{1}\left(x\right),M_{2}\left(x\right),\ldots ,M_{k}\left(x\right)\right)\;\;\;\;\;\;\;\;\;\;\\ g_{k}^{T}\left(M_{1}\left(x\right),M_{2}\left(x\right),\ldots ,M_{k}\left(x\right),\rho \left(x,u\right)\right) \end{array} \end{array}\right]$$

If the system of Equation (33) is correct, then one can immediately find the function $\rho \left(x_{1},x_{2},\ldots ,x_{k},u\right)$, which determines the transformation by static feedback, linearizing the control system B. Therefore, the procedure for determining the function $\rho \left(x_{1},x_{2},\ldots ,x_{k},u\right)$ using the matrix Equation (33) is a procedure for synthesizing the linearizing transformation that determines the controller. Moreover, if the matrix Equation (33) is correct, then h$\left(y_{1},y_{2},\ldots ,y_{k}\right)=M_{1}^{-1}\left(y\right)\;$is, by definition, a flat output of the control system B. Thus, the described procedure for synthesizing the linearizing transform is also a procedure for determining the flat output of nonlinear control systems and, at the same time, a test for whether a given nonlinear control system is a flat system of type A.

## 4. An Example of Applying the Controller Synthesis Algorithm to a DDWMR

### 4.1. Kinematic Model of a DDWMR and Class I Transformations

**Figure 8:**
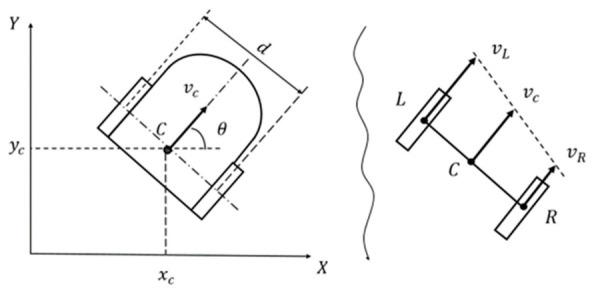
Geometric parameters of the robot and its kinematic variables. Point C (the nominal geometric center of the robot) lies at the intersection of the robot’s longitudinal axis of symmetry and the axis connecting the centers of the drive wheels.

Consider the kinematic model of a DDWMR moving on a horizontal plane. As state variables fully describe the robot’s position and orientation, it is convenient to choose the coordinates $\left(x_{c}.y_{c}\right)$ of point C in a fixed “world” Cartesian coordinate frame XY together with the robot’s heading angle θ (see Figure 8). Let $v_{c}$ denote the velocity vector of point C and let $v_{L}$ and $v_{R}$ denote the velocity vectors of the centers of the left wheel (point L) and the right wheel (point R), respectively.

We may regard points L and R as the endpoints of a rigid, thin rod (the imaginary “rear axle” of the robot), whose midpoint is point C. All three points therefore belong to the same rigid body—the robot chassis. As it is well known [36,37], for the velocity vectors $v_{A}$ and $v_{B}$ of any two points A and B of a rigid body, the relation given in (36) holds, where Ω is the angular velocity vector of the body.

(34) $$v_{A}-v_{B}=\Omega \times r_{AB}$$

The robot’s angular velocity vector Ω is perpendicular to the floor plane. Accordingly, if we denote the unit vector normal to the plane by p, then the angular velocity vector may be written as Ω=ωp. In what follows, when referring to the angular velocity of the robot, we shall mean the scalar quantity ω, whose sign determines the direction of rotation of the robot’s chassis in the horizontal plane. In the absence of lateral slip of the drive wheels, the vectors $v_{L}$ and $v_{R}$ are perpendicular to the axle LR. As follows from Equation (34), applied to points L and R, the projections of $v_{L}$ and $v_{R}$ onto the axis $Y^{*}$ satisfy equality (35) at any moment of time.

(35) $$v_{L}-v_{R}=d\omega$$

Using Equation (35), it is straightforward to show that the vector $v_{c}$ is parallel to the vectors $v_{L}$ and $v_{R}$. Consequently, the velocity of point C can be written as $v_{c}=v_{c}n$, where $n=\left(\cos\theta ,\sin\theta \right)$ is the unit vector aligned with the robot’s heading direction. Furthermore, from the general relation (34) applied to points R and C, equality (36) follows.

(36) $$v_{C}-v_{R}=\left(\frac{d}{2}\right)\omega$$

Equations (35) and (36) together imply Equation (37).

(37) $$\begin{array}{cc} \omega =\frac{1}{d}\left(v_{L}-v_{R}\right) & v_{C}=\frac{v_{L}+v_{R}}{2} \end{array}$$

The above statement regarding the direction of the vector $v_{c},$ together with the fact that the time derivative of the heading angle, $\dot{\theta },$ is precisely the angular velocity of the robot’s chassis, leads to the system of differential Equation (38).

(38) $$\left\{\begin{array}{c} {\dot{x}}_{c}=v_{C}\cos\theta \\ {\dot{y}}_{c}=v_{C}\sin\theta \\ \dot{\theta }=\omega \end{array}\right.$$

Equation (38) can be regarded as the dynamical equations of a control system with state space $Y=R^{2}\times S^{1}$ and control space $W=R^{2}$, representing the simplest kinematic model of a differential-drive wheeled mobile robot. The state vector of this model, q∈Y, describing the robot’s position and orientation on the plane, is given by $q=\left({\left[x_{c},y_{c}\right]}^{T},\theta \right)$, and the control vector w∈W is $w={\left[v_{c},\omega \right]}^{T}$. In the absence of longitudinal slip, the velocities of the wheel centers $v_{L}$ and $v_{R}$ satisfy relations (39), where r is the radius of the robot’s wheels and ${\dot{\varphi }}_{R}$ and ${\dot{\varphi }}_{L}$ are the angular velocities of the right and left drive wheels, respectively (with $\varphi_{R}$ and $\varphi_{L}$ denoting the wheel shaft rotation angles, which may be measured using incremental rotary encoders).

(39) $$\begin{array}{cc} v_{L}=r{\dot{\varphi }}_{L} & v_{R}=r{\dot{\varphi }}_{R} \end{array}$$

We transform the kinematic robot model A into a control system Q with state space $X=R^{2}\times M$, where $M=R\times S^{1}$ and control space $U=R^{2},$ whose state vector has the form $x=\left(p,\left(u,\theta \right)\right)$, and whose dynamics are given by (40).

(40) $$\left\{\begin{array}{c} {\dot{p}}_{x}=u\cos\theta \\ \begin{array}{c} {\dot{p}}_{y}=u\sin\theta \\ \dot{u}=a_{t}\\ \dot{\theta }=\omega \end{array} \end{array}\right.$$

The system Q is obtained by transforming the control signal $u={\left[u_{1},u_{2}\right]}^{T}\in U$ of the robot’s kinematic model (where $u_{1},u_{2}$ are aliases for $v_{c},\omega$) into the control signal $w={\left[w_{1},w_{2}\right]}^{T}$ of the system Q (where $w_{1},w_{2}$ are aliases for $a_{t},\omega$), analytically defined by Equation (41).

(41) $$w_{1}={\dot{u}}_{1}\;\;\;\;w_{2}=u_{2}$$

This transformation is an example of a class I control system transformation, whose definition is given below. Recall that a control system $\dot{x}=f\left(x,u\right)$, $\left(x,u\right)\in X\times U$ is equivalent to the system $\dot{x}=f\left(x,u\right)$, $\dot{u}=v$, since both systems are described by the same infinite-dimensional system $\left\langle X\times U\times \right.\left.R_{m}^{\infty },F\right\rangle$, where the tangent vector field is defined by $F\left(x,u,u^{1},\ldots \right)=\left(f\left(x,u\right),u^{1},u^{2},\ldots \right)$. Let the control signal of a control system be m-dimensional and written as $u={\left[u_{1},u_{2},\ldots ,u_{m}\right]}^{T}$, and let $L_{j}$ be a linear operator whose action on the signal $u\left(t\right)$ is defined by Equation (42).

(42) $$L_{j}u\left(t\right)={\left[u_{1}\left(t\right),\ldots ,{\dot{u}}_{j}\left(t\right),\ldots ,u_{m}\right]}^{T}$$

**Figure 9:**
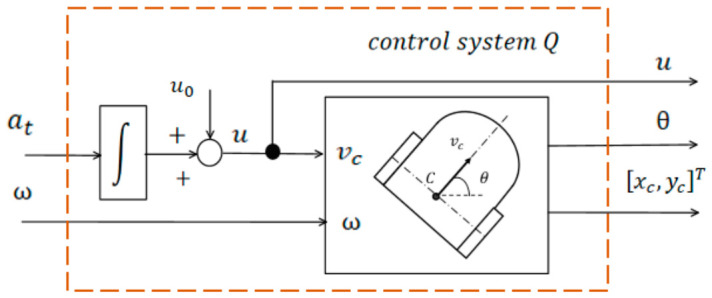
Hardware-implementable scheme for transforming the DDWMR kinematic model A into the control system Q.

It is evident that the control system $\dot{x}=f\left(x,u\right)$, $L_{j}\left(u\right)=v$ is equivalent to the original control system $\dot{x}=f\left(x,u\right)$, $\left(x,u\right)\in X\times U$. We shall refer to such transformations of control systems as class I transformations. The scheme of the transformation of the original kinematic model of DDWMR in the form of a unicycle into the control system Q is shown in Figure 9.

### 4.2. Application of the Class C Linearizing Transformation Synthesis Procedure for the Extended State Space DDWMR Kinematic Model

In this example, the nonlinear control system B is the control system Q with the state space $Y=R^{2}\times M$ and the control space $W=R^{2}$. Any state of the system y∈Y is represented by state variables $y_{1}\in R^{2}$ и $y_{2}\in M$, which are physically interpreted as the positions of the robot’s center, $y_{1}={\left[p_{x},p_{y}\right]}^{T},$ in the Cartesian coordinate system XY given on the plane, as well as the polar coordinates of the velocity vector $y_{2}=\left(u,\theta \right)$, where θ also represents the heading angle of the mobile robot. Accordingly, we will denote the components of the state space $Y=Y_{1}\times Y_{2}$ as $Y_{1}=R^{2}$ and $Y_{2}=R\times S^{1}$. The Equation (40) of the dynamics of the control system Q can be written as a system of differential Equation (43) for the state variables $y_{1}$ and $y_{2}$.

(43) $$\begin{array}{c} {\dot{y}}_{1}=g_{1}\left(y_{2}\right)\\ {\dot{y}}_{2}=w^{T} \end{array}\;\;\;\;y_{1}\in Y_{1},y_{2}\in Y_{2,}$$

where $w^{T}={\left[a_{t},w\right]}^{T}$.

Note that the equations of the dynamics of the control system B in the form (43) have the characteristic structure of the form (32), which indicates the potential possibility of linearizing this system by applying a coordinate transformation of C. The structure of the state space Y and the form of the equation of the dynamics of the system B in the form (38) determine the choice of the state space X of the control system A, the region D in the space $R^{4}=R^{2}\times R^{2},$ and the coordinate transformation M:D→D. As the state space X of the control system A, it is natural to take the Cartesian product X=Position×Velocity, where $Position=R^{2}$ and $Velocity=R^{2}$, while physically interpreting the state variables $x_{1}\in Position$ and $x_{2}\in Velocity$ as a radius vector and as a velocity vector, specifying the current position and velocity of a point moving along a plane. With this natural physical interpretation of the state variables, it is convenient to introduce the aliases $z=x_{1}$ and $v=x_{2}$ to name them. Having identified the planes along which the wheeled robot and the imaginary material point move, we define the first component of the mapping $M_{1}:X\to Y_{1}$ by Formula (44):

(44) $$\forall z_{x},z_{y},v_{x},v_{y}\in R\;M_{1}\left(z_{x},z_{y},v_{x},v_{y}\right)=\left(z_{x},z_{y}\right)\in Y_{1}$$

Since physically, the second component $\left(y_{2}\right)$ of the state y of the system B is a representation of the velocity vector of the robot’s center in polar coordinates, the identification of the velocity vector of the point v with the velocity of the robot’s center defines two possible types of mapping $M_{2}:X\to Y_{2}$, the first of which is represented by Formula (45).

(45) $$\forall z_{x},z_{y},v_{x},v_{y}\in RM_{2}\left(z_{x},z_{y},v_{x},v_{y}\right)=\left(\sqrt{v_{x}^{2}+v_{y}^{2}},atan\left(\frac{v_{y}}{v_{x}}\right)\right)\in Y_{2}$$

This variant corresponds to the chart $\left(V,\phi \right)\;$of a smooth manifold Y, in which the support of the chart $V=Y_{1}\times M^{+}$ is a smooth submanifold of Y, where $M^{+}$ is a part of the cylinder $Y_{2}=R\times S^{1}$, given in coordinates $\left(U,\theta \right)$ by the expression $M^{+}=\left\{\left(u,\theta \right)\in Y_{2}|u>0\right\}$. The diffeomorphism $\phi :{Y_{1}\times M}^{+}\to D$ is defined by two components: the identity mapping ${id:Y}_{1}\to Position$ and the restriction of the mapping $\pi :Y_{2}\to Velocity$ given by Formula (46) to the subset $\left(Velocity/\left\{V0\right\}\right),$ where V0 is the zero-velocity vector:

(46) $$\begin{array}{cc} v_{x}=u\cdot \cos\theta & v_{y}=u\cdot \sin\theta \end{array}$$

Accordingly, the area D is defined $as\;D=Position\times \left(Velocity/\left\{V0\right\}\right)$. We will denote the control signal vector u of the control system A as a and define its dynamic equation as (47), i.e., we will define A as a trivial linear system $T^{2,2}:$

(47) $$\begin{array}{c} \dot{p}=v\\ \dot{v}=a \end{array}\;\;\left(p,v\right)\in X,a\in U$$

Let us carry out the procedure for synthesizing a linearizing transformation described in the previous section, which is simultaneously a test for the existence of such a transformation, for the considered local description of the control systems A and B. By direct calculation, we get the Jacobian matrix $J_{M}\left(x\right)$ of the coordinate transformation M:D→D, defined by the components $M_{1}:D\to Y_{1}$ (44) and $M_{2}:D\to \left(Velocity/\left\{V0\right\}\right)$ (45). The calculation result is presented by Formula (48) for the dependence of the Jacobian matrix$\;J_{M}\left(x\right)$ of the coordinate transformation M:D→D on the state $x=\left(z_{x},z_{y},v_{x},v_{y}\right)$ of the system A:

(48) $$J_{M}\left(z_{x},z_{y},v_{x},v_{y}\right)=\left[\begin{array}{ccc} 1 & 0 & \begin{array}{cc} 0 & \;\;\;\;\;\;0 \end{array}\\ 0 & 1 & \begin{array}{cc} 0 & \;\;\;\;\;0 \end{array}\\ \begin{array}{c} 0\\ 0 \end{array} & \begin{array}{c} 0\\ 0 \end{array} & \begin{array}{c} \begin{array}{cc} \frac{v_{x}}{v} & \;\;\;\;\frac{v_{y}}{v} \end{array}\\ \begin{array}{cc} \frac{v_{y}^{2}}{v^{2}} & -\frac{v_{x}^{2}}{v^{2}} \end{array} \end{array} \end{array}\right]\;$$

where, for brevity, the notation $v=\sqrt{v_{x}^{2}+v_{y}^{2}}$ is introduced.

It is easy to verify by direct calculation the validity of Formula (49) for the function $g_{1}$ from the equation of the dynamics of the control system B in the form (43) and the function $g_{2}\left(y,w\right)=w^{T},\;$implicitly defined by this equation:

(49) $$\begin{array}{cc} \forall x\in X\;g_{1}(M^{-1}\left(x)\right)={\left[v_{x},v_{y}\right]}^{T}\; & g_{2}(M^{-1}\left(x),\rho \left(x,u\right)\right)=\rho^{T}\left(x,a\right) \end{array}$$

where $\rho^{T}\left(x,a\right)={\left[\rho_{1}\left(x,u\right),\rho_{2}\left(x,u\right)\right]}^{T}$.

Then, the matrix equation for the synthesis of the linearizing transformation (28) is represented by Formula (50):

(50) $$\left[\begin{array}{ccc} 1 & 0 & \begin{array}{cc} 0 & \;\;\;\;\;\;0 \end{array}\\ 0 & 1 & \begin{array}{cc} 0 & \;\;\;\;\;0 \end{array}\\ \begin{array}{c} 0\\ 0 \end{array} & \begin{array}{c} 0\\ 0 \end{array} & \begin{array}{c} \begin{array}{cc} \frac{v_{x}}{v} & \;\;\;\;\frac{v_{y}}{v} \end{array}\\ \begin{array}{cc} \frac{v_{y}^{2}}{v^{2}} & -\frac{v_{x}^{2}}{v^{2}} \end{array} \end{array} \end{array}\right]\left[\begin{array}{c} v_{x}\\ v_{y}\\ \begin{array}{c} a_{x}\\ a_{y} \end{array} \end{array}\right]=\left[\begin{array}{c} v_{x}\\ v_{y}\\ \begin{array}{c} \rho_{1}\left(x,a\right)\\ \rho_{2}\left(x,a\right) \end{array} \end{array}\right]$$

The only solution to Equation (50) is represented by Formula (51):

(51) $$\begin{array}{c} \rho_{1}\left(x,a\right)=\frac{v_{x}\cdot a_{x}+v_{y}\cdot a_{y}}{\sqrt{v_{x}^{2}+v_{y}^{2}}}\\ \rho_{2}\left(x,a\right)=\frac{v_{y}\cdot a_{x}-v_{x}\cdot a_{y}}{v_{x}^{2}+v_{y}^{2}} \end{array}$$

These functions define a linearizing transformation of class C, linearizing the kinematic model of the DDWMR. The function $d\left(x,a\right)$, corresponding to Formula (51) defining the DDWMR trajectory tracking controller, is determined by two components, $d_{a_{t}}$ and $d_{\omega }$, which have the forms (52) and (53), respectively:

(52) $$d_{a_{t}}\left(\left(u,\theta \right),\left(a_{x},a_{y}\right)\right)=a_{x}\cos\theta +a_{y}\sin\theta$$

(53) $$d_{\omega }\left(\left(u,\theta \right),\left(a_{x},a_{y}\right)\right)=\frac{a_{x}\sin\theta -a_{y}\cos\theta }{u}$$

**Figure 10:**
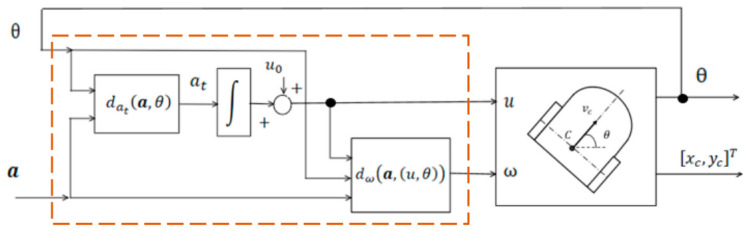
Linearization scheme for the kinematic model of the DDWMR.

The corresponding scheme of the internal nonlinear loop of the trajectory control controller of a wheeled robot is shown in Figure 10.

The corresponding unified trajectory controller for flat systems of type A will be a controller for the forward movement of a wheeled robot, in which the sign of the projection of the robot’s velocity vector onto its longitudinal axis u must remain positive, i.e., at any moment t of the robot’s movement $u\left(t\right)>0$.

If we take another version of the mapping $M_{2}:X\to Y_{2}$, described by Formula (54),

(54) $$\forall z_{x},z_{y},v_{x},v_{y}\in R\;M_{2}\left(z_{x},z_{y},v_{x},v_{y}\right)=\left(-\sqrt{v_{x}^{2}+v_{y}^{2}},atan\left(\frac{v_{y}}{v_{x}}\right)+\pi \right)\in Y_{2}$$

and carry out a similar procedure for synthesizing a trajectory tracking controller, we obtain a similar linearization scheme for the backward motion of the robot. Thus, it is sufficient to change the definition of the function $d_{\omega }$ (54) to definition (55) in order to obtain a trajectory tracking controller that allows one to set the trajectories of the robot’s movement, for which the robot moves both forward and backward:

(55) $$d_{\omega }\left(\left(u,\theta \right),\left(a_{x},a_{y}\right)\right)=\left\{\begin{array}{c} \frac{a_{x}\sin\theta -a_{y}\cos\theta }{u}\\ 0,\;\;otherwise\; \end{array}\right.,\;if\;\left|u\right|>\varepsilon$$

where ε>0 is a small constant, the value of which determines the accuracy of following a given trajectory at low robot speeds.

**Figure 11:**
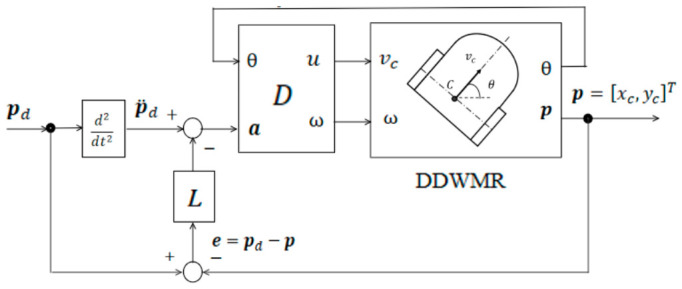
A unified trajectory tracing controller for flat type A systems, synthesized for DDWMR. The robot center position, described by the vector $p={\left[x_{c},y_{c}\right]}^{T}$, is the flat output of the DDWMR kinematic model.

The corresponding DDWMR trajectory tracking controller scheme used for the simulation is shown in Figure 11.

## 5. Analysis of the Resulting Control System

A significant difference between the trajectory control system for a mobile wheeled robot proposed in this paper and most currently known systems is the nature of the trajectory tracking error dynamics. As will be shown below, the trajectory tracking error dynamics (regardless of the cause of the error—whether it is a disturbance or a consequence of a mismatch between the initial conditions and the reference trajectory) are completely determined by the design of the two-channel linear controller included in the trajectory control circuit. As will be shown below, only two types of linear controllers—PD and PID controllers—can be used in the trajectory control system. To simplify the analysis, we will consider the simpler case of a PD controller. The PD controller corresponds to a linear operator U, whose action on an arbitrary signal specified by a smooth function $f\left(t\right)$ is described by the formula $\forall t\;Uf\left(t\right)=k_{d}\dot{f}\left(t\right)+k_{p}f\left(t\right)$, where $k_{d}$ and $k_{p}$ are the controller tuning coefficients. Accordingly, the trajectory following error $e\left(t\right)=\left[e_{1}\left(t\right),e_{2}\left(t\right)\right]$ is determined by solutions of the Cauchy problems for differential Equation (51), which describe the dynamics of its components $e_{1}\left(t\right)$ and $e_{2}\left(t\right)$ and the initial conditions specified by Formulas (56) and (57):

(56) $${\ddot{e}}_{k}\left(t\right)-k_{d}{\dot{e}}_{k}\left(t\right)-k_{p}e_{k}\left(t\right)=0$$

(57) $$\begin{array}{cc} e_{k}\left(0\right)=e_{k0} & {\dot{e}}_{k}\left(0\right)= \end{array}e_{k0}^{\prime },$$

where $k\in \left\{1,\;2\right\}$ is the integer index of the one-dimensional control channel and $e_{k0}$ and $e_{k0}^{\prime }$ are real constants defining the initial deviation of the robot’s center position and velocity from the desired trajectory.

The criterion of system stability then takes the form of conditions (59) imposed on the roots of characteristic Equation (58):

(58) $$\lambda^{2}-k_{d}\lambda -k_{p}=0$$

(59) $$\left\{\begin{array}{c} Re\left(\lambda_{1}\right)<0\\ Re\left(\lambda_{2}\right)<0 \end{array}\right.$$

It is convenient to specify the values of the tuning coefficients by the values of the roots of the characteristic equation using obvious Formula (60) that describe the relationship between the values of the coefficients of the quadratic equation and its roots:

(60) $$\begin{array}{cc} k_{d}=\lambda_{1}+\lambda_{2} & k_{p}=-\lambda_{1}\cdot \lambda_{2} \end{array}$$

It is most reasonable to set $\lambda_{1}=\lambda_{2}=-a$, where a>0 is a real parameter that determines the tuning coefficients of the PD controller according to Formula (61):

(61) $$\begin{array}{cc} k_{d}=-2\cdot a & k_{p}=-a^{2} \end{array}$$

In this case, the general solution of the differential Equation (56), describing the dynamics of the tracking error components, has the form (62):

(62) $$e\left(t\right)=C_{k}^{1}te^{-at}+C_{k}^{2}e^{-at}$$

where the constant coefficients $C_{k}^{1}$ and $C_{k}^{2}$ are determined by the initial conditions (57), according to Formula (63):

(63) $$\begin{array}{cc} C_{k}^{2}=a\cdot e_{k}^{\prime }+e_{k0} & C_{k}^{2}=e_{k0} \end{array}$$

By increasing the value of the parameter *a*, one can (theoretically) achieve an arbitrarily high rate of convergence of the trajectory to the reference trajectory. Certainly, in practical implementation, the values of the PD controller’s setting coefficients are limited by both the maximum power of the wheeled robot’s motors and its design parameters (the position of the robot’s center of gravity, etc.), as well as by the physical parameters of the external environment (primarily the coefficient of friction of the tires on the surface). Two important aspects should be highlighted. First, the proposed DDWMR trajectory control method does not impose any requirement for the initial system state to be close to the desired trajectory. Consequently, the controller remains effective even under substantial deviations from the reference path, which may arise due to external disturbances. Second, the proposed trajectory controller design does not use time-varying control lows, and the controller settings are not tied to the selected desired trajectory. In order to confirm the above statements and to experimentally evaluate the performance characteristics of the proposed trajectory control controller design, a computer simulation of the trajectory control system was conducted, the results of which are presented in the next section. As a reference for comparison, we will use the simulation results of the differential flatness-based DDWMR trajectory control system proposed in paper [50]. The trajectory control method proposed in that paper is based on the use of time-varying control lows, which allows for achieving a high convergence rate (the speed of convergence to a predetermined desired trajectory) and high trajectory tracking accuracy. In addition, paper [50] contains a detailed description of the simulation parameters, which makes it convenient to use the results presented in the article for comparative research.

### Simulation Results

Three different reference trajectories were used in the tests.

Trajectory 1 “Sinusoid”.

The reference trajectory of the robot center was specified by Equations (64) and (65) on the time interval [0, 20] s:

(64) $$x_{ref}\left(t\right)=v_{x}\cdot t$$

where $v_{x}=1\;m{\cdot s}^{-1}$.

(65) $$y_{ref}\left(t\right)=A\sin\left(\omega t\right)$$

where A=1 m and $\omega =0.5\;\mathrm{rad}\cdot s^{-1}$.

**Figure 12:**
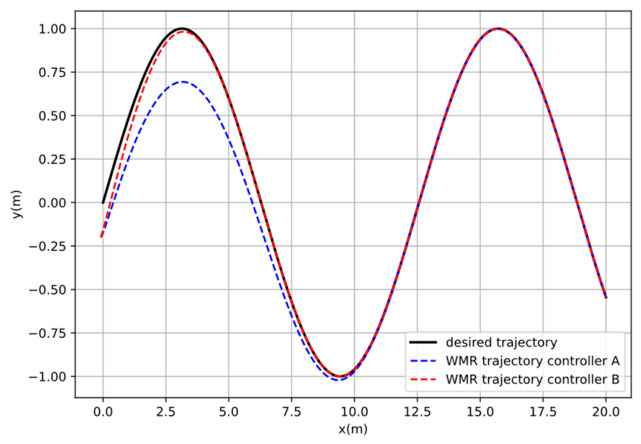
Spatial trajectories 1.

A simulation was performed with two variants of a linear controller from one initial posture ($x_{0},y_{0},\theta_{0})$ = (−0.1 m, −0.2 m, 0.0 rad), with initial velocity $u_{0}=0.2\;m{\cdot s}^{-1}$. In both variants, a two-channel PD controller was used with coefficients $k_{d}=-1.2,$ $k_{p}=0.36$ (for trajectory controller A) and $k_{d}=-3.2$ and $k_{p}=2.56$ (for trajectory controller B). The results are shown in Figure 12.

**Figure 13:**
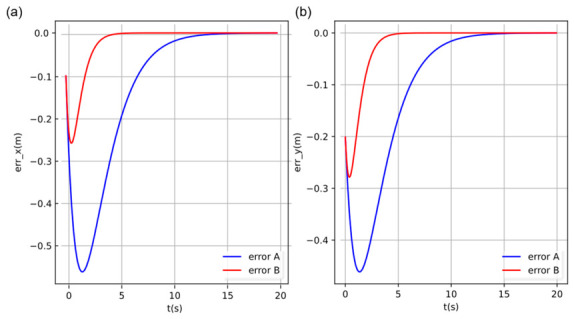
Tracking errors for channel X (**a**) and channel Y (**b**) of trajectories 1.

The tracking error graphs for controllers A and B for the X and Y channels are shown in Figure 13.

**Figure 14:**
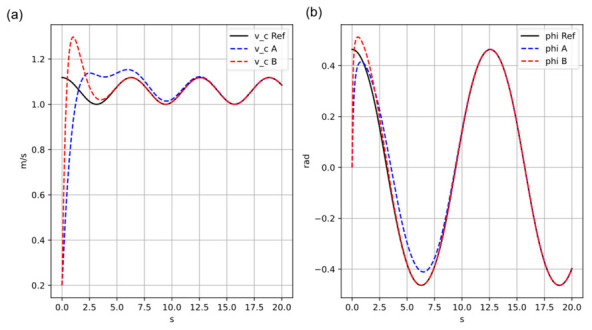
The robot center velocity (**a**); the heading angle (**b**) of trajectories 1.

The graphs of the velocity of the robot center and the heading angle are shown in Figure 14.

Trajectory 2 “Figure-eight”.

The reference trajectory of the robot center was specified by Equation (66) on the time interval [0, 20] s

(66) $$\begin{array}{cc} x\left(t\right)=-2.0\cdot \cos\left(\omega \cdot t\right) & y\left(t\right)=\sin\left(\omega \cdot t\right) \end{array}$$

where $\omega =\frac{2\pi }{T}\;rad\cdot s^{-1}$ and T=20 s.

**Figure 15:**
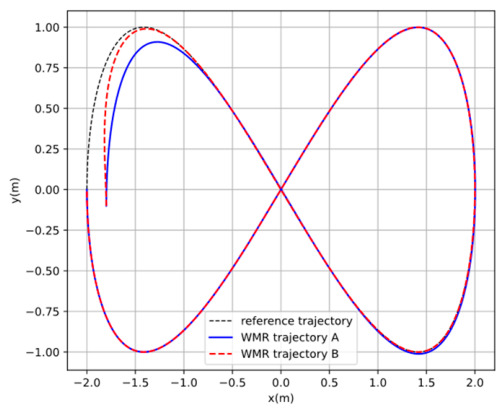
Spatial trajectories 2.

A simulation was performed with two variants of a linear controller from one initial posture ($x_{0},y_{0},\theta_{0})$ = (−1.8 m, −0.1 m, $\frac{\pi }{2}$ rad), with initial velocity $u_{0}=0.5\;m{\cdot s}^{-1}$. In both variants, a two-channel PD controller was used with coefficients $k_{d}=-1.2,$ $k_{p}=0.36$ (for trajectory controller A) and $k_{d}=-3.2$ and $k_{p}=2.56$ (for trajectory controller B). The results are shown in Figure 15.

**Figure 16:**
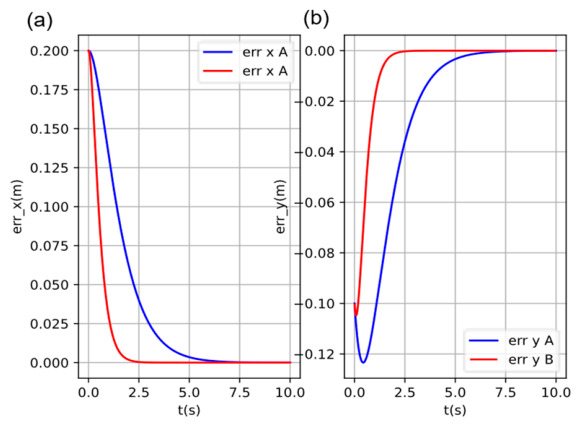
Tracking errors for channel X (**a**) and channel Y (**b**) of trajectories 2.

The tracking error graphs for controllers A and B for the X and Y channels are shown in Figure 16.

**Figure 17:**
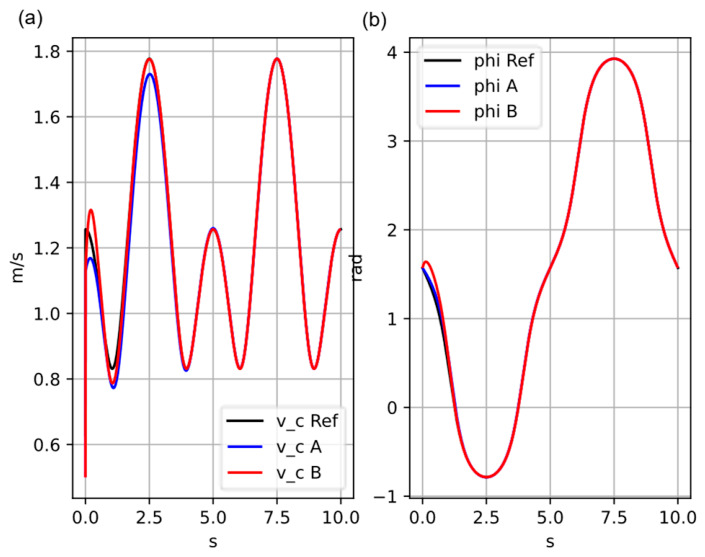
The robot center velocity (**a**); the heading angle (**b**) of trajectories 2.

The graphs of the velocity of the robot center and the heading angle are shown in Figure 17.

Trajectory 3 “Rectangle with smoothed edges” (sharp turns).

The reference trajectory of the robot center was specified in polar coordinates by Equation (67) on the time interval [0, 10] s.

(67) $$\begin{array}{cc} {r\left(t\right)=R\cdot \left({\left|\cos\left(\omega t\right)\right|}^{15}+{\left|\sin\left(\omega t\right)\right|}^{15}\right)}^{-\frac{1}{16}} & \varphi \left(t\right)=\omega t \end{array}$$

**Figure 18:**
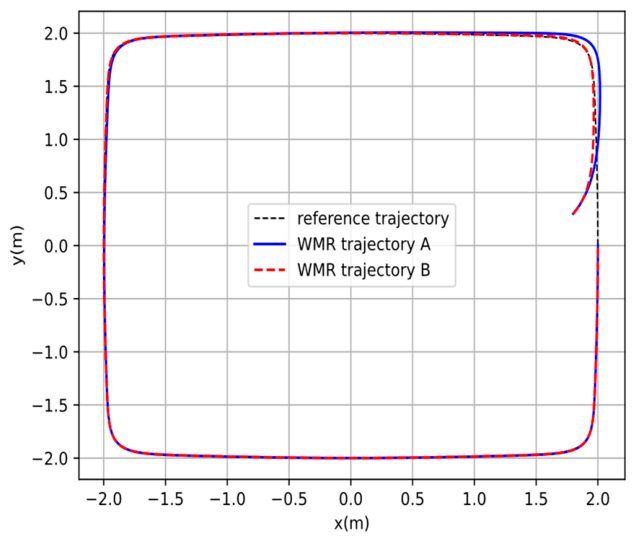
Spatial trajectories 3.

A simulation was performed with two variants of a linear controller from one initial posture ($x_{0},y_{0},\theta_{0})$ = (1.8 m, −0.3 m, 0/95 rad), with initial velocity $u_{0}=1.0\;m{\cdot s}^{-1}$. In both variants, a two-channel PD controller was used with coefficients $k_{d}=-1.2,$ $k_{p}=0.36$ (for trajectory controller A) and $k_{d}=-3.2$ and $k_{p}=2.56$ (for trajectory controller B). The results are shown in Figure 18.

**Figure 19:**
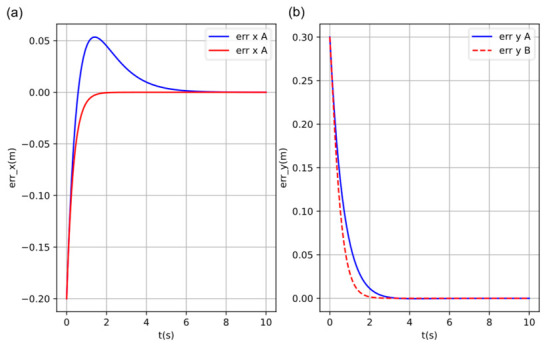
Tracking errors for channel X (**a**) and channel Y (**b**) of trajectories 3.

The tracking error graphs for controllers A and B for the X and Y channels are shown in Figure 19.

**Figure 20:**
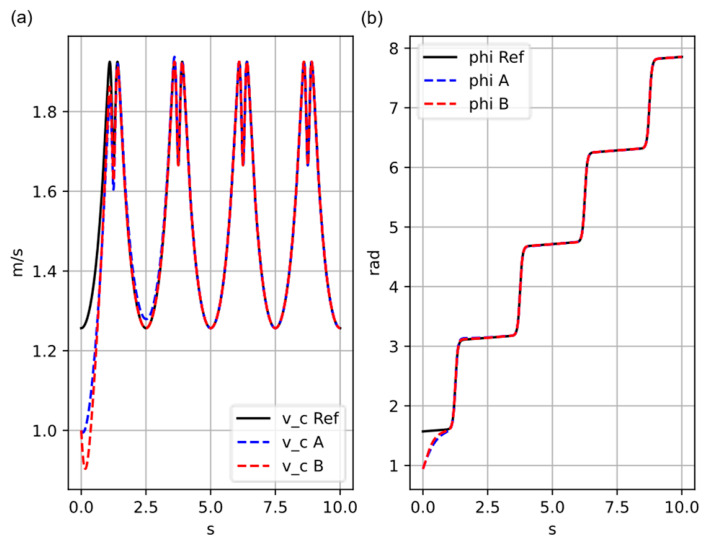
The robot center velocity (**a**); the heading angle (**b**) of trajectories 3.

The graphs of the velocity of the robot center and the heading angle are shown in Figure 20.

As can be seen from Figure 13, Figure 14, Figure 16, Figure 17, Figure 19 and Figure 20, in the absence of disturbances, the tracking error for the forward motion controller described in this article depends solely on the settings of the robot’s linear controller. When analyzing the simulation results, a comparison was made between the theoretically calculated dependence of the tracking error on the Xi and Y channels and the experimental data. As expected, a complete match was obtained between the experimental and theoretical results. We consider such calculations as a test for the correctness of the controller implementation for the simulation. To conduct comparative studies, we used the experimental results presented in articles [50,51]. In particular, we used the desired trajectory for which the authors of article [50] conducted experiments. The desired trajectory is given by Formula (68) for components over t=20 [s]:

(68) $$\begin{array}{c} p_{dx}\left(t\right)=\left(3.75\cdot 10^{-4}\right)\cdot t^{3}-\left(1.125\cdot 10^{-2}\right)\cdot t^{2}+\left(0.15\right)t\\ p_{dy}\left(t\right)=\left(-3\cdot 10^{-4}\right)\cdot t^{3}+\left(9\cdot 10^{-3}\right)\cdot t^{2} \end{array}$$

The initial state of the robot is determined by the initial position $p_{0}={\left[p_{x0},p_{y0}\right]}^{T}$ and two scalar quantities: the initial value of the heading angle $\theta_{0}$ and the initial value $u_{0}=u\left(0\right)$ of the quantity $u\left(t\right)$ (heading speed) (recall that the functions $\theta \left(t\right)$ and $u\left(t\right)$ determine the dependence of the velocity vector of the robot’s center on time $v_{c}\left(t\right)={\left[v_{cx}\left(t\right),v_{cy}\left(t\right)\right]}^{T}$ according to the formulas $v_{cx}\left(t\right)=u\left(t\right)\cos\left(\theta \left(t\right)\right)$ and $v_{cy}\left(t\right)=u\left(t\right)\sin\left(\theta \left(t\right)\right)$).

**Figure 21:**
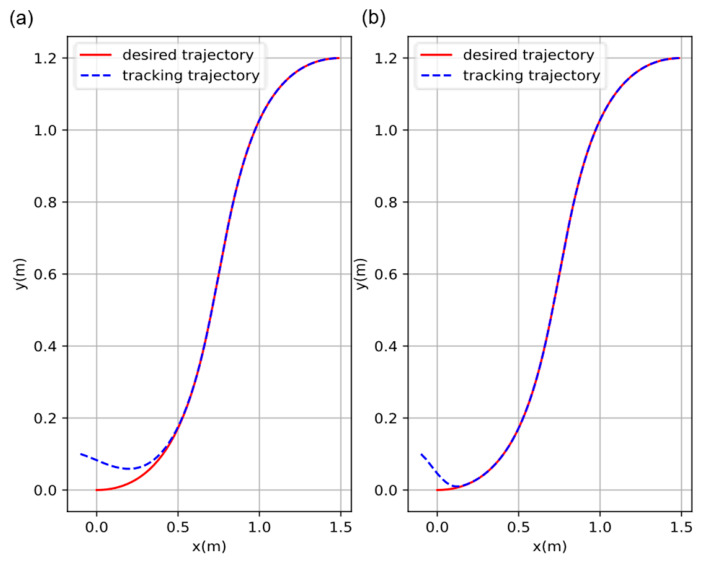
Desired trajectory and tracking trajectory: trajectory tracking system A (**a**); trajectory tracking system B (**b**).

Thus, the initial state of the robot can be represented as a set of four scalar quantities $\left[x_{0},y_{0},u_{0},\theta_{0}\right]$. The same set of quantities was used to define the initial state of the DDWMR trajectory control system in the kinematic model in article [51]. In order to be able to conduct a comparative analysis of two differential flatness-based trajectory control systems, we will use the initial state $\left[x_{0},y_{0},u_{0},\theta_{0}\right]=\left[-0.1,\;0.1,\;0.5,\;-0.5\right]$, which was used by the authors of article [51] when simulating the operation of the DDWMR trajectory control system controller they proposed. To demonstrate the dependence of the performance of the trajectory tracking controller proposed in this work on the design of the linear controller included in the external linear loop of the system, we present simulation results for two trajectory control systems that differ only in the settings of the two-channel PD controllers included in their composition. For the first system, which we will call system A, we will choose the values of the tuning coefficients $k_{p}=-1.44$ and $k_{d}=-2.4$, corresponding to the value of the parameter a=1.2, and for the second system, B, we will choose the coefficients $k_{p}=-36.0$ and $k_{d}=-12.0$, corresponding to a=6.0. Figure 21 shows the graphs of the spatial tracking trajectories of systems A and B for the desired trajectory and initial state specified above.

**Figure 22:**
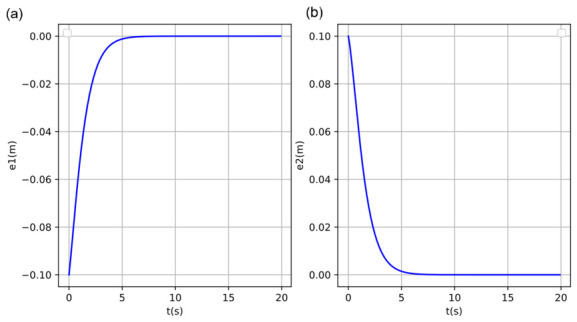
Tracking error $e\left(t\right)={\left[e_{1}\left(t\right),e_{2}\left(t\right)\right]}^{T}$ of system A: tracking error $e_{1}$ (**a**); tracking error $e_{2}$ (**b**).

Figure 22 shows the tracking error graphs for system A.

Note that the graphs of the components of the trajectory following error$\;e_{1}\left(t\right)$ and $e_{2}\left(t\right)$ obtained as a result of the simulation ideally coincide with the graphs of the dependences of the components of the trajectory following error on time $e_{1}^{*}\left(t\right)$ and $e_{2}^{*}\left(t\right)$, calculated using Formulas (62) and (63), the analytical expressions for which are presented by Formulas (69) and (70), respectively:

(69) $$e_{1}^{*}\left(t\right)=-exp\left(-1.2\cdot t\right)\cdot \left(0.07t+0.1\right)$$

(70) $$e_{2}^{*}\left(t\right)=exp\left(-2\cdot t\right)\cdot \left(\left(-0.12\right)\cdot t-0.1\right)$$

**Figure 23:**
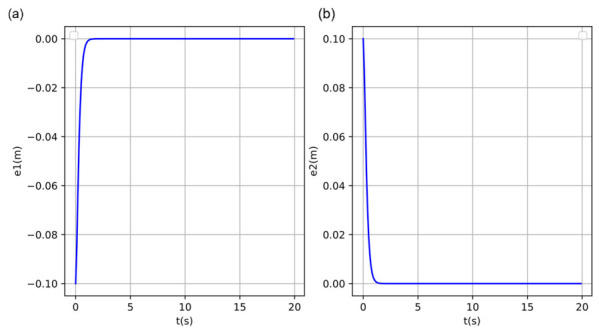
Tracking error $e\left(t\right)={\left[e_{1}\left(t\right),e_{2}\left(t\right)\right]}^{T}$ of system B: tracking error $e_{1}$ (**a**); tracking error $e_{2}$ (**b**).

The graphs obtained during the simulation for the components of the trajectory tracking error for system B are presented in Figure 23.

**Figure 24:**
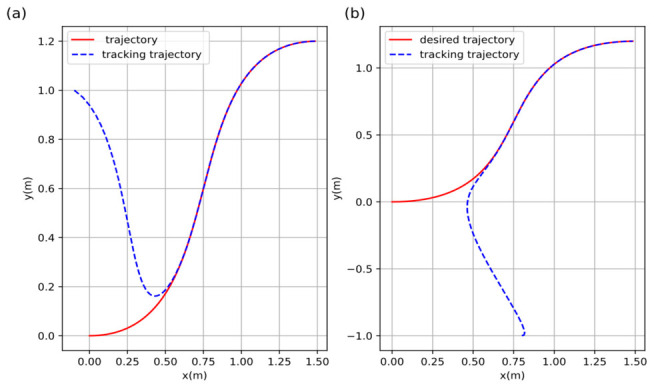
Desired trajectory given by Equation (61) and tracking trajectories for different initial states: initial state $\left[x_{0},y_{0},u_{0},\theta_{0}\right]=\left[-0.1,\;1.0,\;0.05,\;-0.5\right]$ (**a**); initial state $\left[x_{0},y_{0},u_{0},\theta_{0}\right]=\left[0.8,-1.0,\;0.05,\;0.3\right]$ (**b**).

By comparing the graphs shown in Figure 22 and Figure 23 with similar graphs presented in article [52], it is easy to see that the convergence rate of system A is significantly higher than that of the DDWMR trajectory tracking controller described in paper [51]. Clearly, the rate of convergence to a given trajectory for this controller depends on the settings of the linear regulator and is also limited by the technical characteristics of the robot and the physical parameters of the environment. Accordingly, it makes sense to compare not the convergence rates of two adjustable trajectory control controllers but the ease of regulation and the nature of the limitations imposed by the controller design on the conditions of its correct operation. Figure 24 shows graphs illustrating the operation of the trajectory control system of a mobile wheeled robot proposed in this work for large initial deviations from the given trajectory.

**Figure 25:**
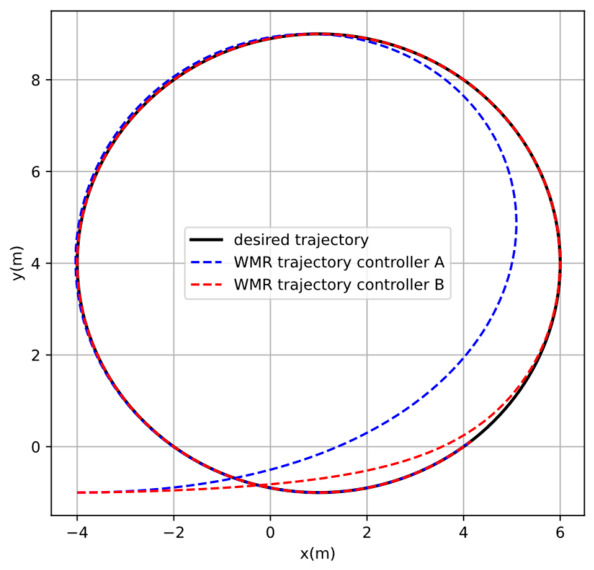
The trajectory tracking of the circle.

In conclusion, we present the simulation results for a circular trajectory and the initial position that were used to test the trajectory control method for a wheeled mobile robot proposed by the authors of [53,54]. In ref. [52], the desired spatial trajectory in the form of a circle of radius R = 5 m centered at a point with coordinates (1, 4) [m], along which a mobile robot should move at a constant speed $u=5\;\frac{m}{s},$ was used to compare the trajectory control controllers for a wheeled mobile robot proposed in refs. [53,54]. In both works, the initial posture $\left[x_{0},y_{0},\theta_{0}\right]$ of the WMR was set as $\left[-4,-1.0\right],$ and the initial velocity of the robot $u_{0}$ was set as $u_{0}=u=5\;\frac{m}{s}$ (which corresponds to the initial position $\left[x_{0},y_{0},u_{0},\theta_{0}\right]=\left[-4,-1.5,\;0\right]$). Figure 25 shows the simulation results for this circular trajectory and initial position for the two trajectory controllers A and B defined above.

When comparing the simulation results shown in Figure 25 with the results presented in paper [52], it becomes clear that controller A outperforms both controllers proposed in refs. [53,54], which were simulated in paper [54].

## 6. Discussion

First, it is important to discuss some aspects of the software and hardware implementation of the proposed DDWMR trajectory control system. The proposed trajectory control system for a mobile robot contains two nested control loops: an outer linear control loop and an inner nonlinear control loop. The outer loop uses position feedback, while the inner loop uses static feedback on a single parameter—the robot’s heading angle. Since the flat output of both the kinematic and dynamic DDWMR models is the position of the robot’s center of gravity (the point C, located midway between the centers of the drive wheels), differential flatness-based trajectory control methods for this type of wheeled robot often require measuring not only the current coordinates of the robot’s center of gravity but also several first-order time derivatives of the flat output—often the velocity and acceleration of the robot’s center of gravity. As a rule, to control the DDWMR kinematic model, it is sufficient to measure the current coordinates and velocity vector of the robot’s center of gravity with sufficient accuracy and data update rate. Note that in addition to differential flatness-based methods of trajectory control of WMRs, a number of other methods also require measuring the speed of the robot’s center.

Thus, one of the distinguishing features of the proposed DDWMR trajectory tracking controller design is the set of physical quantities whose measurements are necessary for its implementation. Another distinguishing feature of the trajectory tracking controller described in this paper is the absence of restrictions on the input data of the trajectory tracking controller. Note that two-layer designs are generally common in the field of trajectory control; the wheeled mobile robot controller described in paper [52] serves as an example of such a design. The upper-layer controller is implemented using a linear time-varying model predictive control (LTV-MPC) scheme, while the lower-layer controller is realized via a radial basis function neural network-based proportional–integral–derivative (RBFNN–PID) algorithm. The main advantage of this two-layer control architecture is its ability to achieve accurate reference path tracking while maintaining vehicle stability. However, such a tracking control strategy is characterized by high computational complexity.

The proposed trajectory controller is essentially a single controller, and there are no restrictions on its input data. However, there are other important reasons why a two-layer design with a kinematic control upper layer is often chosen. Measuring robot posture is the primary navigation task for mobile robots, and every autonomous mobile robot, regardless of its control system design, is equipped with some form of a navigation system. Sensor fusion and processing of data from various sensors are often used to measure posture. Therefore, the update rate of the navigation system is often significantly lower than that of the sensors used in the low-layer control system, which stabilizes the robot.

On the other hand, the speed and accuracy of the sensors used to generate feedback signals are key factors determining the characteristics and design of any control system, and the tasks solved by a low-layer control system require high-speed sensors. In the trajectory control method proposed in this paper, the internal nonlinear feedback loop can, in some sense, be considered as a low layer of a similar two-layer system, in the sense that orientation measurement (course angle measurement) requires a higher data update rate than position measurement in the external linear control loop. We emphasize that the problem of accurately and rapidly measuring the robot orientation has been extensively studied, as precise orientation information plays a key role in the design of tracking control systems for nonholonomic WMRs. Consequently, a number of effective and well-established solutions to this problem are available in the literature. The main difference between position measurement and velocity measurement is that MEMS sensors can directly measure both orientation and velocity, i.e., the angular velocity vector. A MEMS gyroscope outputs angular velocity, and the data can be used to calculate the robot’s orientation.

However, this sensor is affected by accumulated errors. In contrast, an accelerometer and a magnetometer measure the Earth’s gravitational and magnetic fields, respectively, thereby providing an absolute reference for orientation. Nevertheless, these sensors are subject to high levels of noise. Data fusion techniques make it possible to mitigate the high noise levels inherent in MEMS sensor data while preserving the high performance of the resulting composite measurement system. An example of such a solution is the MEMS-based sensor-based orientation measurement subsystem described by Tsai et al. in ref. [52], in which the data fusion algorithm developed by the authors allowed for estimating a single orientation through the optimal data fusion of gyroscope, accelerometer, and magnetometer measurements. The above considerations suggest that the DDWMR trajectory control method proposed in this paper offers certain engineering advantages over both known differential flatness-based trajectory tracking methods and a number of other common trajectory control methods.

The proposed trajectory tracking method is closely related to the well-known CTC technique for serial manipulators, also referred to as inverse dynamics control. At the conceptual level, both approaches rely on transforming a nonlinear plant via static feedback linearization into an equivalent trivial linear controllable system. As a result, the control problem for an *n*-dimensional nonlinear system is reduced to the control of *n* independent one-dimensional decoupled linear subsystems. The resulting trajectory tracking architectures are structurally identical: in both cases, a nonlinear inner loop performs linearization, while a linear outer loop implements standard tracking laws using classical linear controllers. Consequently, together with the recognized advantages of CTC, which remains a highly active research area due to its excellent performance characteristics [41,42,43], the proposed method naturally inherits the limitations that are intrinsic to static-feedback linearization.

A key drawback of CTC type methods is their limited robustness to model uncertainty: even small discrepancies between the real plant and the mathematical model used inside the linearizing controller can lead to noticeable degradation of tracking performance. In this study, a simple kinematic DDWMR model was deliberately chosen to ensure transparency of the approach and ease of simulation. Nevertheless, our simulation experiments confirm the expected behavior: the performance of the proposed controller is sensitive to parameter mismatches, similarly to classical CTC. This phenomenon is well-documented in the CTC literature, where modern research directions focus on adaptive and robust extensions, including the use of machine learning-based compensation mechanisms [28]. If we consider the application of this general approach to more complex plants (a well-known example is the CTC for sequential manipulators), it becomes clear that the trajectory tracking controller essentially contains a model of the controlled object. Deviations in the values of the model parameters embedded in the controller from their actual values can have a significant negative impact on control accuracy.

Since the trajectory tracking architecture proposed in this paper consists of two nested loops—a nonlinear inner loop that linearizes the plant and an outer loop that performs trajectory tracking of the linearized system—the outer loop can incorporate a wide range of modern control techniques, including robust, adaptive, optimal, or MPC-based controllers. This is a fundamental difference from the most existing flatness-based methods for DDWMRs, which rely on approximate linearization in the neighborhood of a predefined reference trajectory (exact feedforward linearization). Such schemes require a sufficiently accurate match between the initial state of the robot and the nominal initial condition of the reference trajectory, and these may exhibit performance loss or even instability in case the robot starts significantly away from the desired path.

By contrast, the method proposed here avoids these limitations because it performs plant linearization, not trajectory linearization. In other words, it is the nonlinear plant itself that is converted into an equivalent linear controllable system, rather than the entire trajectory tracking closed loop. As highlighted earlier, current research trends increasingly focus on adaptive flatness-based techniques and on the application of MPC, optimal control and hybrid control methods to flat systems [15,16,25,26,27]. Notably, many of these techniques become particularly effective and easier to implement when applied to differentially flat systems [28], making the proposed architecture compatible with a broad class of advanced control methods.

Another characteristic distinction of the proposed approach is that it yields a SISO (single-input–single-output) control structure for the linearized system. In contrast, most existing DDWMR trajectory tracking methods rely on multivariable controllers with coupled control channels [27]. The SISO nature of the resulting linear system significantly simplifies controller design, tuning, and implementation, providing a conceptually straightforward yet highly effective solution.

Obviously, due to environmental phenomena such as wind, wheel slippage, actuator saturation, sensor noise, etc., not taken into account in the modeling, there will always be a difference between the reality and the mathematical model describing the movement of a wheeled mobile robot. Regardless of which particular trajectory control method is used in the design of the trajectory control system, the question inevitably arises: how can this trajectory control method, when applied to a WMR, ensure accurate tracking of the desired trajectory despite the presence of uncertainties? For differential flatness-based methods, which include the WMR trajectory control method proposed in this paper, this issue is particularly acute, for reasons specific to this group of methods, briefly listed below. As noted in the very informative review by Abadi et al. in paper [34], to date, a limited number of methods have been presented in the literature addressing robustness issues of flatness systems. The most interesting approach is that which the authors of paper [34] applied to the problem of developing a robust control system based on the differential flatness-based trajectory control system DDWMR. Abadi et al. presented a new cascade control strategy that utilized a combination of flatness, active disturbance suppression, and an improved SMC strategy that eliminates the chattering phenomenon characteristic of this control method. The chattering phenomenon was mitigated by applying the boundary layer method within SMC, while some reduction in robustness that occurred with this modification was compensated by applying the active disturbance rejection control (ADRC) technique. This deep modification of the differential flatness-based trajectory tracking controller, which demonstrated high performance in real experiments, indicated one of the possible ways to radically improve the method proposed in this paper.

Currently, a lot of attention is paid to the issues of the influence of various factors on mobile robot energy consumption: methods of power consumption estimation and modeling of energy consumption for WMRs of various types [55,56,57,58,59]. In particular, for a DDWMR, which is the object of study in this paper, information on these issues can be found in refs. [56,57]. At the same time, the issue of the influence of the controller on WMR energy consumption is little covered in the literature, and there is a handful of works dedicated to this question, for example, papers [58,59]. This state of affairs is quite natural. As research results [55,56] show, the most significant factors influencing power consumption are payload and acceleration, which have an obvious physical justification and are intuitively understandable. Accordingly, it may seem that for WMRs in general and DDWMRs in particular, after design decisions, the main factor influencing the robot’s energy consumption is robot trajectory planning rather than the controller. Indeed, trajectory planning significantly influences energy consumption, and many studies have been devoted to energy-efficient trajectory planning (e.g., [59]). Under near-ideal conditions (no wheel slippage, skidding, or faults of drives), the controller’s impact on energy consumption will be relatively minor. The impact of the above factors leads to deviation of the robot from the reference trajectory. After restoring the normal operating mode, the robot, which briefly deviates from the reference trajectory, again returns to it, and the speed of convergence of the real trajectory of the robot to the specified one and the real trajectory of the robot during the exit to the specified trajectory are determined by the robot controller. Stefek et al. [58] conducted a direct comparison of mobile robot motion controllers and noticed that controllers with low energy consumption lacked accuracy due to their smooth control actions and vice versa—accurate controllers were shown to have a higher consumption. A special feature of the trajectory tracking controller presented in this paper is the complete predictability of tracking error dynamics and the ability to control it using the settings of the linear controller included in the outer feedback loop. When simulating the controller’s operation, it is easy to observe that with an increase in the convergence rate, the average and maximum values of the radial component of the acceleration vector of the robot’s center increase on average. This is a physically predictable effect, and the influence of controller settings on the trajectory convergence rate is demonstrated by the simulation results presented in the paper. Also, based on the graphs of the dependence of the robot’s center velocity module (see Figure 14, Figure 17 and Figure 20), when moving along the same trajectory with different settings of the trajectory tracking controller, one can see a correlation between the trajectory convergence rate and the average and peak values of the radial acceleration of the robot’s center. Note that a simultaneous simulation of two controllers, A and B, was performed on the same trajectories, where A is a controller that provides fast convergence and B is a controller that provides a relatively slow convergence rate. In addition, based on the graphs of the robot’s center velocity magnitude while moving along the same trajectory with different settings of the trajectory tracking controller, a correlation can be seen between the trajectory convergence rate and the average and peak values of the robot’s radial acceleration. Furthermore, reducing the trajectory convergence rate reduces the likelihood of undesirable phenomena, such as wheel slippage and skidding. Generally, the required convergence rate, which, in our case, directly corresponds to the accuracy of the trajectory tracking, is determined by the reference trajectory. For the trajectory tracking controller presented here, it is possible to set the minimum convergence rate sufficient for a given application, corresponding to minimal power consumption.

## 7. Further Perspectives

Future research is expected to proceed in two directions. First, the proposed linearization-based trajectory tracking method can be extended from the kinematic DDWMR model to its full dynamic model, as well as to other classes of mobile robots, including balancing robots, aerial vehicles, and multi-degree-of-freedom robotic platforms. Such generalizations would enable a broader assessment of the method’s applicability to complex nonlinear robotic systems. Second, an important avenue for further work is the development of robust and adaptive extensions of the proposed controller. Given its sensitivity to model uncertainties—an inherent limitation shared with classical computed torque control—our future research will focus on designing robust, adaptive, and learning-enhanced compensation mechanisms that preserve the advantages of the linearized structure while improving resilience to parameter variations and external disturbances. We are currently beginning to organize a series of full-scale experiments with the DDWMR local positioning system, which allows robot position determination, and we also plan to use the PlatypOUs mobile robotic platform [60] to test the developed methods.

## 8. Conclusions

This work has presented a unified trajectory tracking controller for a special type of differential flat control system, which might be referenced as the type A flat systems. Here, this refers to systems whose flat output depends only on the state. A special case of such a unified controller is the CTC controller. The proposed controller retains the most attractive features of the CTC and CTC-like methods. This transformation allows the nonlinear trajectory tracking problem to be reformulated as a classical linear control task for several decoupled linear subsystems, greatly simplifying the controller design while maintaining strong theoretical guarantees. While CTC-like methods are usually associated with second-order mechanical systems, the proposed new method can handle differentially flat systems of arbitrary order, in particular, systems obtained from the original plant model by applying the class I transformation described in the paper (i.e., an extension of the state space of the control system that preserves its trajectory equivalence properties).

The proposed approach inherits principal CTC advantages: transparency of design, explicit control of the closed-loop dynamics, and high tracking accuracy for sufficiently well-modeled systems. Simulation results confirm these benefits: the robot successfully converges to the desired circular trajectory, achieving a maximum transient deviation of 0.28 m, a settling time of approximately 120 s, and a steady-state mean tracking error below 0.01 m. These results demonstrate that linearizing the plant itself, rather than the trajectory tracking loop, provides a simple yet powerful mechanism for achieving high-precision tracking in nonlinear mobile robots.

The proposed framework establishes a general and extensible foundation for trajectory tracking control of differentially flat systems and demonstrates strong potential for both theoretical development and practical deployment.

## Data Availability

The data presented in this study are available on request from the corresponding authors.
